# 3D Crystal Construction by Single‐Crystal 2D Material Supercell Multiplying

**DOI:** 10.1002/advs.202411656

**Published:** 2024-11-18

**Authors:** Wenhao Li, Jichuang Shen, Yaqing Ma, Xiang Xu, Han Chen, Lida Yu, Chen Ji, Menglin He, Kezhao Ma, Yiwei Duo, Li Wang, Tongbo Wei, Liping Shi, Muhong Wu, Kaihui Liu, Huaze Zhu, Wei Kong

**Affiliations:** ^1^ Zhejiang University Hangzhou 310027 China; ^2^ School of Engineering Westlake University Hangzhou 310030 China; ^3^ School of Engineering Westlake University Hangzhou 310027 China; ^4^ Zhongke Crystal Materials (Dongguan) Technology Co., Ltd. Dongguan 523000 China; ^5^ Hangzhou Institution of Technology Xidian University Hangzhou 311231 China; ^6^ Research and Development Center for Semiconductor Lighting Technology Institute of Semiconductors Chinese Academy of Sciences Beijing 100083 China; ^7^ Center of Materials Science and Optoelectronics Engineering University of Chinese Academy of Sciences Beijing 100049 China; ^8^ Institute of Physics Chinese Academy of Sciences Beijing 100190 China; ^9^ Institute of Atomic Manufacturing Beihang University Beijing 100083 China; ^10^ Interdisciplinary Institute of Light‐Element Quantum Materials and Research Centre for Light‐Element Advanced Materials International Centre for Quantum Materials Peking University Beijing 100091 China; ^11^ Songshan Lake Materials Laboratory Institute of Physics Chinese Academy of Sciences Dongguan 523781 China; ^12^ State Key Laboratory for Mesoscopic Physics School of Physics Peking University Beijing 100091 China; ^13^ Research Center for Industries of the Future Westlake University Hangzhou Zhejiang 310024 China; ^14^ Zhejiang Key Laboratory of 3D Micro/Nano Fabrication and Characterization Westlake Institute for Optoelectronics Fuyang Hangzhou Zhejiang 311400 China

**Keywords:** 2D/3D integration, 3R‐MoS₂ artificial crystal, high throughput 2D stacking, nonlinear optical (NLO) crystals, second harmonic generation (SHG)

## Abstract

2D stacking presents a promising avenue for creating periodic superstructures that unveil novel physical phenomena. While extensive research has focused on lateral 2D material superstructures formed through composition modulation and twisted moiré structures, the exploration of vertical periodicity in 2D material superstructures remains limited. Although weak van der Waals interfaces enable layer‐by‐layer vertical stacking, traditional methods struggle to control in‐plane crystal orientation over large areas, and the vertical dimension is constrained by unscalable, low‐throughput processes, preventing the achievement of global order structures. In this study, a supercell multiplying approach is introduced that enables high‐throughput construction of 3D superstructures on a macroscopic scale, utilizing artificially stacked single‐crystalline 2D multilayers as foundational repeating units. By employing wafer‐scale single‐crystalline 2D materials and referencing the crystal orientation of substrates, the method ensures precise alignment of crystal orientation within and across each supercell, thereby achieving controllable periodicity along all three axes. A centimeter‐scale 3R‐MoS₂ crystal is successfully constructed, comprising over 200 monolayers of single‐crystalline MoS₂, through a bottom‐up stacking process. Additionally, the approach accommodates the integration of amorphous oxide, enabling the assembly of 3D non‐linear optical crystals with quasi‐phase matching. This method paves the way for the bottom‐up construction of macroscopic artificial 3D crystals with atomic plane precision, enabling tailored optical, electrical, and thermal properties and advancing the development of novel artificial materials and high‐performance applications.

## Graphical abstract

We present a high‐throughput approach for constructing macroscopic 3D superstructures with three‐axis periodicity using single‐crystalline 2D materials. We achieve rapid, bottom‐up stacking to form 200‐layer 3R‐MoS₂ structures and incorporates oxide interlayers for quasi‐phase matching in nonlinear optical crystals. This technique enables atomic layer precision in 3D crystal construction with tailored material properties.

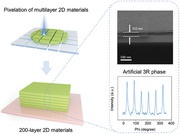

## 1 Introduction

The study of 2D materials has generated immense interest due to their unique properties and the potential for applications not possible with 3D materials.^[^ ^1^, ^2^, ^3^, ^4^ ^]^ A key advantage of 2D materials is their weak van der Waals (vdW) interfaces, which facilitate the stacking of layers without requiring lattice matching.^[^ ^5^, ^6^ ^]^ This characteristic enables the formation of non‐equilibrium structures, potentially leading to novel physical phenomena. For example, when bilayers or trilayers of 2D materials are stacked with a slight twist, moiré patterns emerge, resulting in lateral superlattices with distinctive electronic and optical properties.^[^ ^7^, ^8^, ^9^, ^10^, ^11^ ^]^ While the in‐plane periodic structures have been under intensive investigation, the vertical periodicity in 2D material stacking remains relatively unexplored. The creation of vertical superlattices with atomic precision holds great promise for uncovering new physical phenomena and enabling advanced applications.^[^ ^12^, ^13^, ^14^ ^]^ However, achieving such vertical periodicity presents significant challenges, particularly in terms of controlling the in‐plane crystal orientation and scaling the vertical dimension.

Previous efforts in 2D material stacking have often been based on polycrystalline materials,^[^ ^15^, ^16^, ^17^ ^]^ which lack precise control over the in‐plane orientation due to random rotations of local grains. This randomness impedes the controllable formation of well‐ordered vertical superstructures, leading to the polycrystalline nature of the stacked structure. Conversely, single‐crystalline 2D layers have been employed to construct 2D stacking; however, these methods typically involve mechanical exfoliation from bulk materials, which is low‐throughput and results in irregularly shaped flakes, thus complicating the achievement of vertical scalability.^[^ ^18^, ^19^, ^20^ ^]^ Consequently, the realization of scalable 2D superstructures with controllable long‐range periodicity, both in‐plane and out‐of‐plane, has remained elusive.

## 2 Results

### 2.1 High Throughput 2D Stacking by Supercell Multiplying

Single‐crystalline 2D materials synthesized at the wafer scale via chemical vapor deposition (CVD) have emerged as a highly promising platform for advancing innovations in 2D materials.^[^ ^21^, ^22^, ^23^ ^]^ These materials offer not only exceptional uniformity across large areas but also a well‐defined crystal orientation, enabling precise control of thickness and orientation over stacking. Additionally, the large volumes of material produced through CVD provide ample material volume for constructing thick stacking structures. However, stacking CVD single‐crystalline 2D materials presents two significant challenges. First, the strong adhesion between these 2D materials and their substrates complicates exfoliation, typically requiring the use of polymethyl methacrylate (PMMA) and an interface liftoff in a solvent. This process often results in uncontrolled flotation, making it difficult to accurately determine crystal orientation.^[^ ^22^, ^24^ ^]^ Second, the monolayer‐by‐monolayer stacking process is inefficient, limiting the scalability of these structures in terms of thickness.

**Figure 1:**
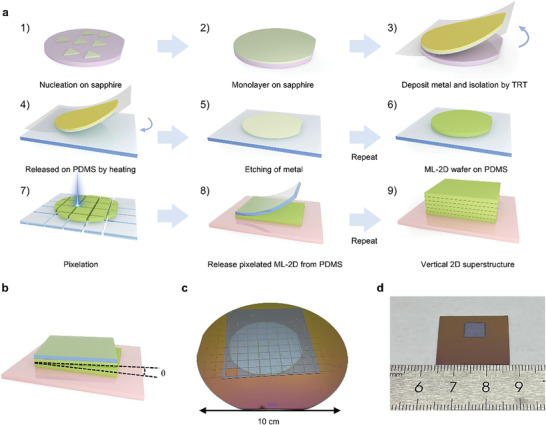
Supercell multiplying approach. a) Scheme of the supercell multiplying approach. Single‐crystalline MoS_2_ is synthesized on sapphire substrate (step 1–2). After Cu deposition, Cu/MoS_2_ is exfoliated using TRT and released on a PDMS thin film via heating (step 3–4). Cu is then removed by wet etching (step 5). By repeating these steps, 10L MoS_2_ is sequentially stacked on PDMS (step 6). The 10L MoS_2_ is then pixelated into rectangle supercells by laser (step 7). Twenty 10L MoS_2_/PDMS supercells are selected and the MoS_2_ in supercells are sequentially released on the target substrate SiO_2_/Si (step 8–9) b) Schematics of orientation alignment. c) Photographs of a pixelated 2‐inch 10L MoS_2_ on PDMS. d) Photograph of a stacked centimeter‐scale 200L 3R‐MoS_2_ transferred onto a SiO_2_/Si substrate_._

To address these challenges, we introduce a 2D supercell multiplying approach, as illustrated in **Figure** **1**a. A vertical 2D superstructure consists of fundamental repeating supercells with specific 2D stacking orders. Thus, we begin by fabricating these repeating artificial supercells in bulk quantities as the basic units for subsequent stacking, thereby ensuring stacking efficiency and consistency across different supercells. To overcome the adhesion of single‐crystalline 2D materials to their substrate (see Figure S1, Supporting Information for the single crystallinity of the MoS_2_), we utilize deposited metal as the adhesive for exfoliation, leveraging the stronger adhesion of metal to the 2D materials (see Methods for details).^[^ ^25^, ^26^, ^27^, ^28^, ^29^ ^]^ The metal/2D bilayer is then attached to a polydimethylsiloxane (PDMS) stamp, followed by metal etching. Repeating these steps results in the formation of a multilayer 2D material (ML‐2D) at wafer scale, with the material type of each layer fully adjustable. Subsequently, a high‐power laser beam is used to pixelate the ML‐2D on the PDMS stamp, and stacking the pixelated ML‐2D supercells enables the rapid vertical scaling. The pixelation process can be further repeated to improve stacking efficiency.

The epitaxial relationship between the 2D materials and their crystalline substrates facilitates alignment across different substrates by using substrate orientation as a reference. For instance, the flats of sapphire wafers, which are normal to the (11$\bar{2}$0) direction, serve as a reliable reference direction for the stacking of wafer scale ML‐2D. During the alignment of different ML‐2D supercells, the angles between the straight edges formed during pixelation determine the twist angles, as depicted in Figure 1b. Therefore, crystal orientation within and across each supercell is ensured. Figure S2 (Supporting Information) illustrates a 2‐inch MoS₂ wafer with ten layers stacked on a PDMS stamp, which was then pixelated and stacked (see Figure 1c). Using this supercell multiplying approach, we successfully constructed a centimeter‐scale MoS₂ superstructure consisting of 200 layers with 0° twisting across each layer and supercell (see Figure 1d).

Our methodology demonstrates exceptional compatibility with a broad spectrum of 2D materials, including large‐area, single‐crystalline, CVD‐grown 2D materials, such as monolayer MoS₂, WS₂, and MoSe₂ on sapphire and SiO_2_ substrates (refer to Figures S3 and S4, Supporting Information), as well as monolayer graphene and hexagonal boron nitride (h‐BN) grown on metal substrates (see Figure S5a–d, Supporting Information). Beyond monolayer 2D materials, this approach is also applicable to CVD‐grown multilayer 2D materials (see Figure S5e–g, Supporting Information). This compatibility notably broadens the selection of fundamental units for 2D stacking, thereby enhancing both the design flexibility and scalability of stacking structures (Table S1, Supporting Information provides a detailed discussion on the method's versatility). Additionally, these 2D superstructures can be transferred onto target substrates with varying surface conditions while preserving excellent surface integrity (see Figure S6, Supporting Information). Compared to the conventional layer‐by‐layer PDMS method, our process minimizes residue since only the topmost layer contact the PDMS during multilayer stacking, facilitating an ultra‐clean 2D‐2D interface and good coupling between adjacent layers (see Figures S7–S9 and Notes S1 and S2, Supporting Information). The cross‐sectional HADDF‐STEM indicate deposition and etching process do not damage 2D materials (see Figure S10, Supporting Information). Photoluminescence and Raman spectra further confirm no defects or strain was induced transfer process (see Figure S11, Supporting Information). The Field‐effect transistors based on the transferred MoS₂ exhibit reliable electrical properties (see Figure S12, Supporting Information).

### 2.2 Bottom‐Up Construction of Macroscopic 3R‐MoS₂ Artificial Crystal

Among the various polytypes of MoS₂, the 2H phase, characterized by inversion symmetry, is the most thermodynamically stable form found in nature.^[^ ^30^ ^]^ In contrast, the meta‐stable 3R‐MoS₂ phase lacks inversion symmetry, resulting in significant nonlinear susceptibilities, making it particularly promising for optoelectronic and photonic applications.^[^ ^31^, ^32^ ^]^ Recent developments have successfully produced large‐area single‐crystalline multilayer 2D materials,^[^ ^33^, ^34^, ^35^ ^]^ for example thick 3R‐MoS_2_ by CVD.^[^ ^36^ ^]^ However, the grown thick 2D materials lack flexibility in twist angle and composition in each atomic layer during growth, due to requirements for energy minimization. An efficient stacking process that allows for creation of arbitrary twist angles and is compatible with various 2D materials is still needed for the construction of complex superlattices, leveraging the availability of synthesized monolayer or multilayer single‐crystalline 2D materials.

**Figure 2:**
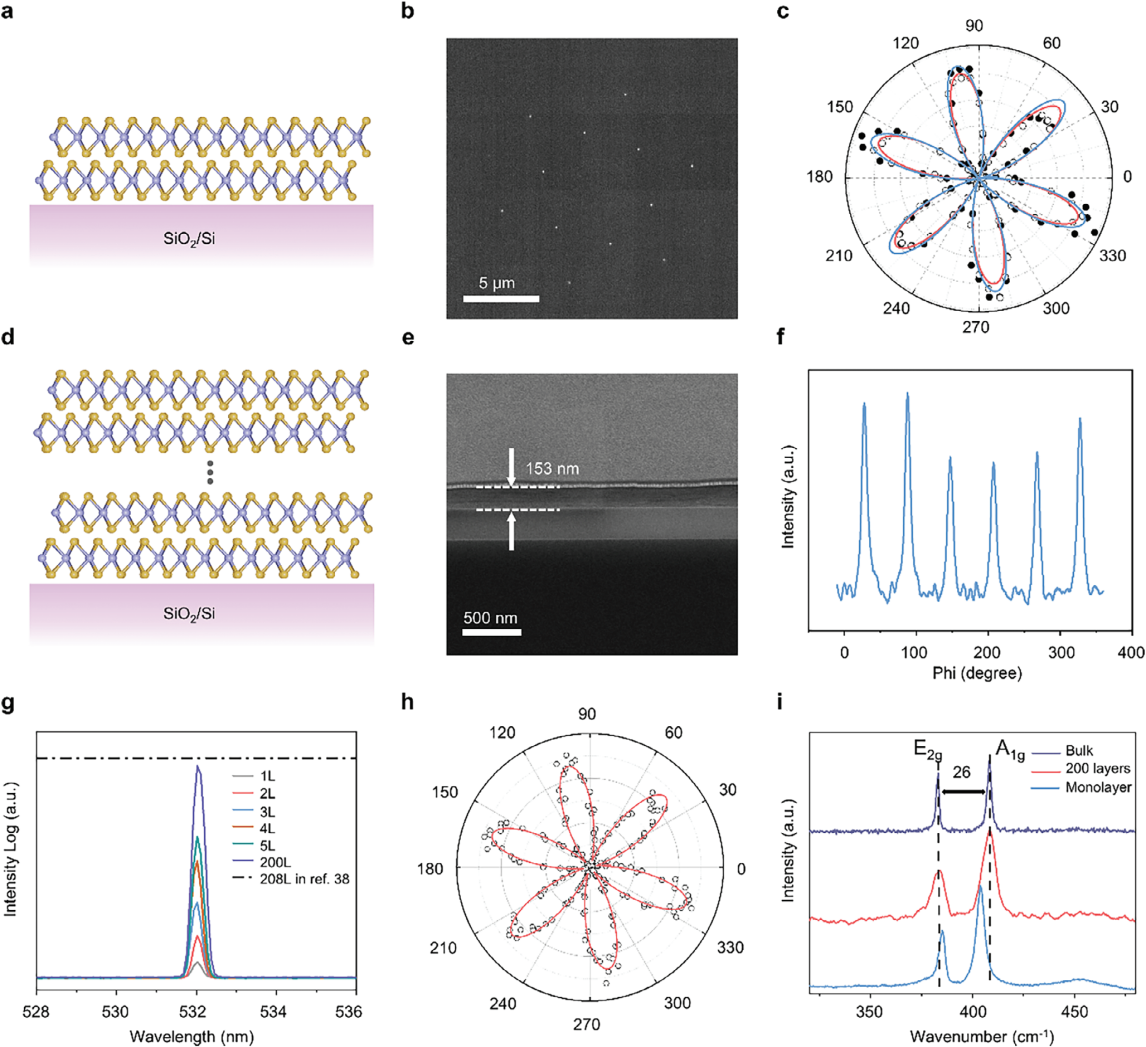
Macroscopic fabrication of the 3R‐MoS_2_. a) Schematics of bilayer 3R‐MoS_2_. b,c), SAED pattern (b) and polarization‐dependent SHG (c) of the bilayer 3R‐MoS_2_. Filled circles and hollow circles are experiment data of each layer. Solid lines are fitting lines. d) Schematics of the 200L 3R‐MoS_2_. e,f) Cross sectional SEM (e), XRD Phi‐scan (f) of the 200L 3R‐MoS_2_. g), SHG intensity of stacked 3R‐MoS_2_ from one to five layers and the 200L 3R‐MoS_2_. h) Polarization‐dependent SHG of the 200L 3R‐MoS_2_. Hollow circles are experiment data and solid line is fitting line. i) Raman spectrum of the 200L 3R‐MoS_2_ compared with monolayer and bulk.

The precise control of orientation alignment of each stacking layer in our approach was first demonstrated in an artificial bilayer 3R‐MoS₂. A schematic diagram of a typical 3R‐phase is shown in **Figure** **2**a, illustrating that adjacent MoS₂ layers have a rhombohedral stacking sequence. This in‐plane orientation alignment was confirmed by selected area electron diffraction (SAED) (see Figure 2b), in which only one set of sharp diffraction patterns with sixfold symmetry is observed. The non‐centrosymmetric 3R stacking sequence was further validated by polarization‐dependent second harmonic generation (SHG) measurements, as depicted in Figure 2c, showing enhanced SHG intensity (see Figure S13 and see Note S3 and Figures S14‐ and S15 (Supporting Information) for details about interlayer alignment).

The supercell multiplying approach enables the efficient fabrication of artificial 3R‐MoS₂, comprising 200 layers corresponding to a thickness of 153 nm (see Figure 2c; Figure S16a, Supporting Information). We employed X‐ray diffraction (XRD) to characterize the crystallinity of our artificial 3R‐MoS₂, which is the standard technique for structural analysis of bulk crystals. The Phi‐scan (see Figure 2f) reveals a single set of sixfold symmetric diffraction patterns corresponding to the MoS₂ (10$\bar{1}$3) plane, indicating a uniform in‐plane orientation throughout the artificial 3R structure (see Figure 2d). Additionally, Figure S16b (Supporting Information) shows a peak at 2*θ* = 13.6°, corresponding to an interplanar spacing of 0.647 nm, which is close to the theoretical value of 0.65 nm.^[^ ^37^ ^]^ These results demonstrate excellent in‐plane and out‐of‐plane periodicity, confirming the single crystallinity of the artificial 3R‐MoS₂ structure.

The artificially assembled bulk MoS₂ exhibits strong SHG intensity, as shown in Figure 2g, which is a characteristic feature of 3R‐MoS₂. Compared to monolayer MoS₂, the enhancement of SHG achieved by our artificial 3R‐MoS₂ is 53 times greater, close to the reported 56 times enhancement for bulk 3R‐MoS₂ synthesized by using chemical vapor transport (CVT).^[^ ^38^ ^]^ Consistent with the second‐order nature of the process, the SHG power in 3R‐MoS₂ shows a quadratic dependence on the average input laser power, as demonstrated in Figure S16c (Supporting Information). No saturation or hysteresis behavior in the SHG profile was observed up to the maximum input power, indicating no laser‐induced material damage. A representative sixfold polarization‐dependent SHG pattern (Figure 2h) reflects the D6h point group of the 3R crystal with broken inversion symmetry. Additionally, our artificial 3R‐MoS₂ exhibits the typical Raman spectrum of bulk 3R‐MoS₂, as shown in Figure 2i, with a significantly larger E_2g_‐A_1g_ separation compared to that of the monolayer.

### 2.3 2D/3D Superlattice Assembling

Our approach further demonstrates the feasibility of van der Waals integration of 3D thin films into 2D superstructures for the construction of 2D/3D superlattices. Specifically, thin‐film oxides introduce additional functionalities to 2D stackings. For instance, high‐κ dielectric oxides, with their wide bandgap and high breakdown characteristics, can enhance gate control in 2D transistors or serve as protective layers to improve material stability.^[^ ^39^, ^40^, ^41^, ^42^, ^43^ ^]^ Unlike traditional oxide deposition, van der Waals integration of oxides preserves the pristine surface of 2D materials due to the non‐destructive nature of the process.^[^ ^44^ ^]^

**Figure 3:**
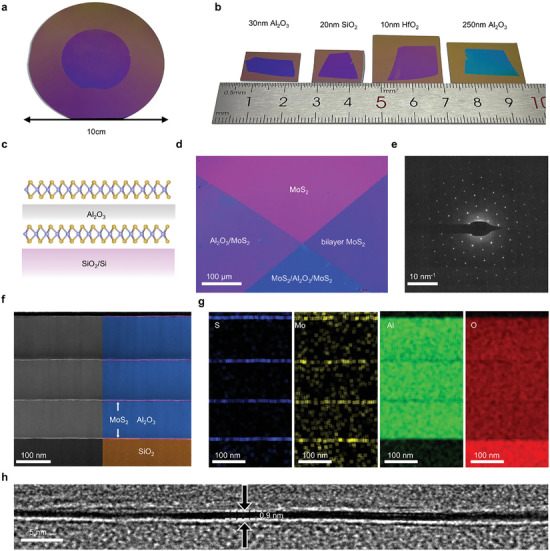
2D/3D superlattice construction. a,b) Photographs of a 2‐inch 30nm Al_2_O_3_ thin film (a) and optical images of various transferred oxides with different thickness (b). The substrates are SiO_2_/Si. c–e) Schematics (c), optical image (d) and SAED (e) of the MoS_2_/Al_2_O_3_/MoS_2_ heterostructure. f,g) False‐color cross sectional STEM (f) and EDS elemental maps (g) of the superlattice with three MoS_2_/Al_2_O_3_ supercells. h) Cross sectional HRTEM of the superlattice.

We show the formation of single‐crystalline 2D and oxide superlattice structures, which are typically unachievable through conventional growth processes due to the challenges in growing large‐area single‐crystalline 2D materials on amorphous oxides. To achieve this, we developed a method to create transferrable wafer‐scale oxide thin films based on deposited oxides on 2D material‐covered sapphire substrates (see Note S4, Supporting Information). Due to the weak van der Waals interface,^[^ ^45^ ^]^ the deposited oxide can be exfoliated as freestanding thin films (see Methods and Figure S17, Supporting Information for details). These films with thickness in submicron range exhibit mechanical properties similar to 2D materials and can be conformally attached to other surfaces using pure van der Waals forces. **Figure** **3**a shows a 2‐inch, 30 nm Al₂O₃ thin film transferred onto a SiO₂/Si substrate, and we demonstrate the compatibility of this process with various oxides of different thicknesses (Figure 3b). Atomic force microscopy (AFM) topographies and optical images of these oxide thin films, shown in Figures S18 and S19 (Supporting Information), reveal clean surfaces without pores or cracks (see Note S5 and Figure S20, Supporting Information for more detail about ALD deposited Al_2_O_3_ on MoS_2_).

We show the integration of oxide thin films with 2D materials at a wafer scale using a 2‐inch MoS₂/Al₂O₃ heterostructure (Figure S21, Supporting Information). A key feature of this approach is the precise control over the orientation of the integrated 2D materials within the 2D/3D stacking, as proven by the MoS₂/20 nm Al₂O₃/MoS₂ sandwich structure depicted in Figure 3c,d. The alignment of the MoS₂ layers on opposite sides of the oxide layer was adjusted to achieve a 0° twisting angle, confirmed by the selected area electron diffraction (SAED) pattern (Figure 3e), which displayed only one set of diffraction patterns.

Vertical scalability was demonstrated through the construction of a superlattice consisting of three 2D/3D supercells, as clearly shown in the cross‐sectional high‐angle annular dark field–scanning transmission electron microscopy (HAADF–STEM) image (Figure 3f). This image reveals four bright horizontal lines corresponding to high atomic number layers (MoS₂), separated by spacer layers (Al₂O₃). Corresponding energy‐dispersive X‐ray spectroscopy (EDS) elemental maps (Figure 3g) precisely depict the stacked layers, with distinct signals for Mo, S, Al, and O that align with the Z‐contrast in the STEM image. The high‐resolution TEM (HRTEM) image (Figure 3h) clearly shows a gap of ≈0.9 nm between the Al₂O₃ layers, with a clean and flat interface. Given that the thickness of a monolayer MoS₂ is ≈0.3 nm,^[^ ^46^ ^]^ and a typical van der Waals gap ranges from 0.3 to 0.5 nm,^[^ ^47^ ^]^ the observed gap between adjacent Al₂O₃ layers should be 0.9–1.3 nm. This consistency suggests the absence of intercalation or contamination residues at the 2D/3D interface.

### 2.4 Assembly of Non‐Linear Optical Crystal Achieving Quasi‐Phase Matching

**Figure 4:**
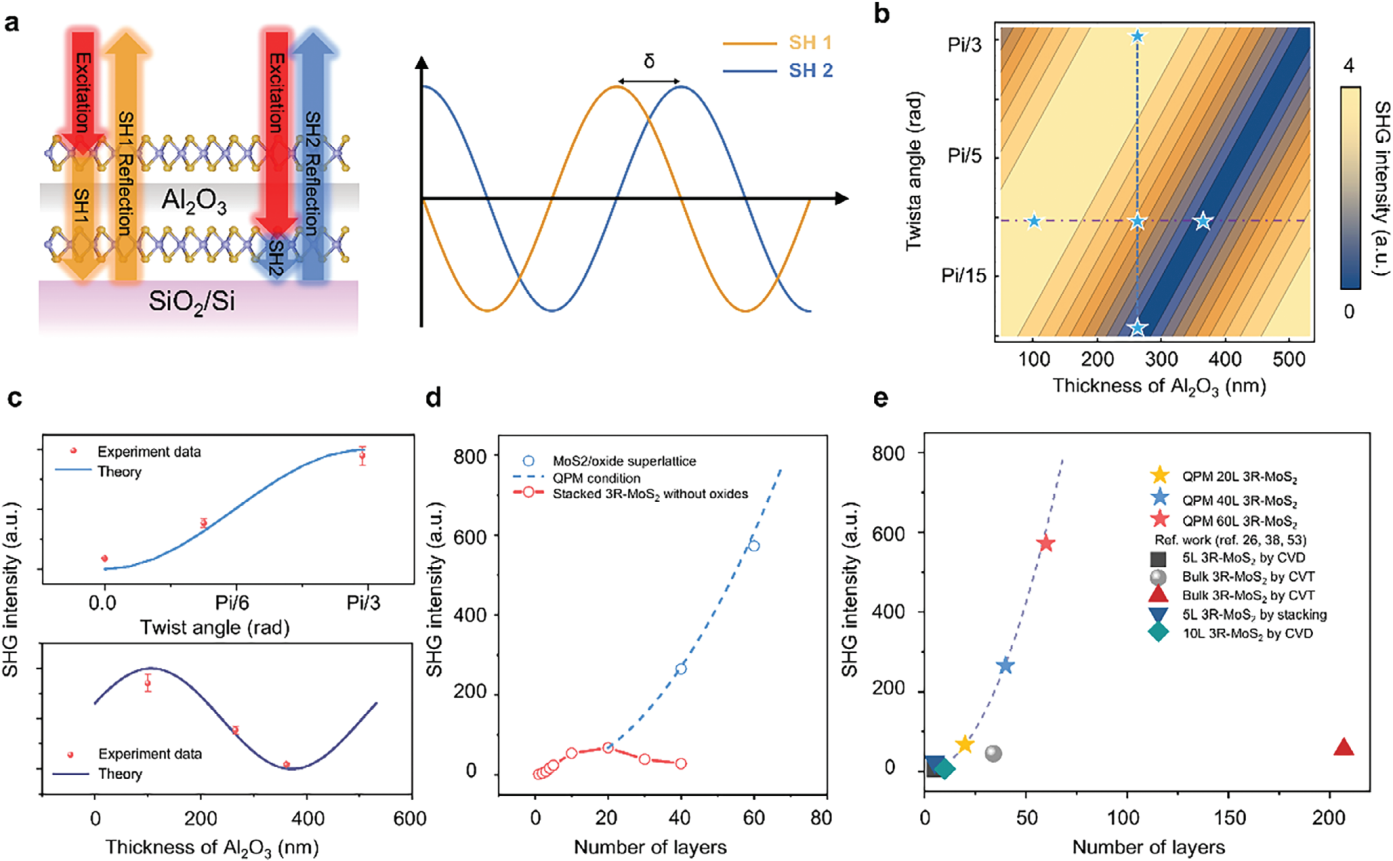
Modulation of SHG and quasi phase matching in 2D/oxides superlattice. a) Scheme of generation and propagation of SH signals in 2D/oxides superlattice (left). Scheme of wave of SH1 and SH2 and their phase difference δ (right). b) Calculation results of SHG intensity based on model illustrated in (a). c) SHG intensity as a function of twist angles with oxide thickness of 266nm (top) and SHG intensity as a function of oxide thickness with the twist angle of 23° (bottom). The blue (purple) solid line is the theoretical data from bule (purple) dashed lines in (b). d) SHG intensity for 3R‐MoS_2_ with (blue circles) and without (red circles) inserted oxide thin films as functions of number of MoS_2_ layers. Dashed blue line is the theoretical intensity in QPM condition. e) Benchmark of SHG intensity in 3R‐MoS_2_ crystals. Dashed line is the theoretical intensity in QPM condition.

In SHG, dispersion causes the fundamental and second harmonic waves to propagate at different phase velocities. This disparity results in phase mismatch, leading to destructive interference of the generated second harmonic waves and significantly reducing the efficiency of SHG. This challenge is a fundamental aspect in the design of SHG optical crystals.^[^ ^48^ ^]^ As illustrated in **Figure** **4**d (red circles), the SHG intensity in the reflection geometry of our stacked 3R‐MoS₂ rapidly increases until the thickness exceeds 21 nm, corresponding to a coherence length of 26 nm or 30 layers of 3R‐MoS₂ (see Note S6, Supporting Information).

Periodic modulation of the nonlinear medium allows for the resetting of the phase relationship between the fundamental and second harmonic waves, thereby compensating for phase mismatch over multiple periods, a process known as quasi‐phase matching (QPM).^[^ ^49^, ^50^, ^51^ ^]^ Previous study has showed a phase‐matching strategy by tuning twist angles of ML 2D materials.^[^ ^48^, ^52^ ^]^ In this study, we introduce a novel strategy to achieve QPM in an artificial 3R‐MoS₂ optical crystal by constructing a periodic 2D/3D superlattice, in which not only twist angles but also 3D materials were induced to tune the optical properties. Figure 4a (left) illustrates a trilayer system where two monolayers of MoS₂ (1L‐MoS₂) are separated by an inserted Al₂O₃ thin film, with a twist angle between the two 1L‐MoS₂ layers. The incident fundamental excitation laser (red vector) irradiates the material vertically from the top. Second harmonic (SH) signals are generated in both the upper (SH1, yellow vector) and lower 1L‐MoS₂ layers (SH2, blue vector) and are reflected at the SiO₂/Si interface, resulting in a phase difference of δ between SH1 and SH2 (Figure 4a, right). This phase difference arises from two factors: the interlayer twist angle and the optical path difference induced by the thickness of the inserted oxide layer. The calculated SHG intensity for this structure, with varying twist angles and Al₂O₃ thicknesses, is presented in Figure 4b (see Note S7 and Figure S22, Supporting Information). The results indicate specific combinations of twist angles and Al₂O₃ thicknesses that lead to QPM. Figure 4c shows the experimental SHG intensities with varied twist angles or oxide thickness for several combinations, as indicated by the stars in Figure 4b. The experimental data show excellent consistency with the theoretical predictions, confirming the achievement of the QPM in this trilayer system (see Figure S23, Supporting Information for QPM scaling up to three supercells).

The effectiveness of the QPM strategy for constructing thick SHG crystals based on ML 3R‐MoS₂ and oxide superlattices is demonstrated by stacking three supercells of ML 3R‐MoS₂/Al₂O₃, as shown in Figure 4d. The SHG intensity of the superlattice with inserted Al_2_O_3_ thicknesses of 266 nm and twist angles of 60° between adjacent ML 3R‐MoS_2_ shows a substantial increase as the number of MoS₂ layers extends to 60, achieving a 573‐fold SHG enhancement compared to a monolayer MoS₂ (see Figure S24, Supporting Information for schemes of superlattices). These SHG increments show strong agreement with the theoretical calculations of the QPM condition (see Note S8, Supporting Information for details).

The benchmark for reflective SHG performance of the artificial QPM 3R‐MoS₂ crystal is depicted in Figure 4e, in comparison with 3R‐MoS₂ crystals either stacked or grown as reported in the literature.^[^ ^26^, ^38^, ^53^ ^]^ Previous stacked MoS₂ samples do not exceed the coherence length, resulting in a limited SHG response. In contrast, while the thickness of bulk‐grown 3R‐MoS₂ can surpass the coherence length, its maximum SHG response is constrained by phase mismatch. Notably, our results demonstrate that 60 layers of 3R‐MoS₂ with QPM achieving an SHG intensity enhancement of ≈573 times, the highest reported in reflection geometry. This remarkable performance underscores the efficiency and robustness of our technique in development of practical advanced photonic materials and devices, where the integration of 2D materials with tunable SHG properties is desired.

Thus, 2D supercell multiplying using single‐crystalline 2D materials enables the construction of scalable, ordered structures with periodicity along all three axes. The properties of these artificial 3D crystals can be precisely tailored by regulating the superstructure, in terms of the atomic composition and crystalline orientation of each fundamental atomic plane. This approach to material design extends beyond enhancing SHG, encompassing the manipulation of other optical properties, for example photonic band structures and chiral optical response. It can also be applied to the design of electrical and thermal properties, highlighting the potential for creating artificial 3D crystals with atomic plane precision, offering opportunities for novel material properties and applications.

## 3 Conclusion

We have developed a 2D supercell multiplying approach to fabricate vertical 2D superlattices using wafer‐scale, single‐crystalline 2D materials synthesized by CVD. This method enables precise control of twist angles and is compatible with both 2D monolayers and multilayers grown on various substrates. It allows for the high‐efficiency fabrication of artificial 2D crystals, achieving a single crystalline 200‐layer‐thick 3R‐MoS₂ structure through bottom‐up stacking, with an SHG response comparable to bulk 3R‐MoS₂. Additionally, we have achieved scalable 2D/3D integration by interfacing 2D materials with oxide thin films. By combining control over 2D twist angles and spacer thickness, we developed an artificial non‐linear optical crystal composed of a 2D/oxide superlattice that achieves quasi‐phase matching (QPM), demonstrating record reflective SHG enhancement with MoS₂. This method paves the way for exploring the assembly of vertical 2D superstructures and designing functional crystals based on 2D material stacking on a macroscopic scale, enabling the precise tailoring of the electrical, optical, and thermal properties of artificial crystals at the atomic plane level.

## 4 Experimental Section

### Growth of TMDCs

The wafer‐scale single‐crystalline MoS_2_, WS_2_, and MoSe_2_ were grown on 2‐inch sapphire wafers (C/A) using a CVD system with a quartz tube furnace (OTF‐1200X, Hefei Kejing Materials Technology). The furnace was divided into three zones with different temperatures. Elemental S powder (15g, purity 99.9%, Sinopharm Chemical Reagent) was placed in a corundum crucible and heated independently to 180 °C in Zone I, carried by a flow of 200 sccm Ar gas. MoO_3_ powder (80mg, purity 99.9%, Sinopharm Chemical Reagent) was heated to 550 °C in Zone II and carried separately by 3 sccm O_2_ and 100 sccm Ar, avoiding contact with the S vapor. The sapphire substrate was placed in Zone III and heated to 950 °C for 40 min for growth.

For WS_2_ growth, a similar three‐zone furnace setup was employed. S powder (15g, purity 99.9%, Sinopharm Chemical Reagent) was heated to 180 °C in Zone I and carried by a flow of 200 sccm Ar and 5 sccm H_2_ gases. The transition metal source WO_3_/NaCl (100/10mg, purity 99.5%, Sigma–Aldrich) was heated to 650 °C in Zone II. The typical substrate temperature in Zone III was maintained at 970 °C, and the growth time was also 40 min.

For MoSe_2_ growth, a similar three‐zone furnace setup was used. Se powder (15g, purity 99.99% Shanghai Aladdin Biochemical Technology) was heated to 250 °C in Zone I and carried by a flow of 30 sccm Ar and 5 sccm H_2_ gases. The transition metal source MoO_3_ (100mg) was heated to 530 °C in Zone II. The typical substrate temperature in Zone III was maintained at 965 °C, and the growth time was 25 min.

### Growth of Graphene and BN

The single‐crystalline graphene was grown on smooth single‐crystalline Cu (111) foils using a CVD system. The foils were placed in the center of a quartz tube furnace (OTF‐1200X, Hefei Kejing Materials Technology). The furnace was initially flushed with Ar gas and then heated to 1050 °C under a flow of 500 sccm Ar and 10 sccm H_2_ for annealing. Subsequently, 1 sccm CH_4_ was introduced as the carbon source and maintained for 6 h. Afterward, the furnace was cooled naturally to room temperature under 500 sccm Ar and 10 sccm H_2_.

The single‐crystalline BN was grown on smooth single‐crystalline Cu (110) foils using a similar CVD system. The foils were placed in the center of the quartz tube furnace (OTF‐1200X, Hefei Kejing Materials Technology). An Al_2_O_3_ crucible containing precursor ammonia borane (97%, Aldrich) was positioned 1 meter away from the Cu (110) foils. During growth, the foils were heated to 1035 °C under a flow of 500 sccm Ar and 50 sccm H_2_. The gas atmosphere was then adjusted to 5 sccm Ar and 45 sccm H_2_ for 3 h. Simultaneously, the precursor in the Al_2_O_3_ crucible was heated to 65 °C using a heating band.

### Transfer of 2D Materials and Fabrication of 200L 3R‐MoS_2_ Crystal

For the transfer and stacking of TMDCs grown on sapphire and SiO_2_, Cu (0.2‐1µm) was deposited on as‐grown TMDC/sapphire by electron beam evaporation (see Figure S25, Supporting Information for the effect of Cu thickness on the exfoliation process). The Cu/TMDC stack was exfoliated from sapphire by thermal release tape (TRT) and was attached to a commercial PDMS film (DC184, Zhongke Advanced Material) with a thickness of 100–300um. Then, TRT was removed by heating at 120 °C on a hot plate, and Cu was etched by ammonium persulfate (50mg mL^−1^). Addition 2D materials could be stacked on PDMS by repeating the above steps. The TMDC stack was then released on target substrate by peeling off PDMS slowly. Compared to 2D materials grown on sapphire and SiO₂, those synthesized on metal substrates, such as BN and graphene on Cu, bond more strongly with the growth substrates. Therefore, for transferring 2D materials grown on a Cu substrate, Ni (0.2–1 µm) and ferric chloride solution (160 mg mL^−1^) were used as the metal layer and etchant, respectively, instead of Cu and ammonium persulfate. The higher adhesion energy with 2D materials provided by Ni facilitates their exfoliation.

For the fabrication of 200L 3R‐MoS_2_, a 10L wafer‐scale 3R‐MoS_2_ was initially stacked on a PDMS thin film, using the previously discussed method. A femtosecond laser (750 fs, 15 W) was employed to pixelate the wafer‐scale MoS_2_/PDMS into twenty 6 × 7 mm rectangular supercells. The twenty supercells were picked up and sequentially released onto a SiO_2_/Si substrate. During this process, twist angles between adjacent 10L MoS_2_ were controlled to be 0°. For the alignment process, a homemade x‐y‐z micromanipulator, a sample stage with rotational adjustment and a long focal length microscope were used. For the alignment of pixelated supercells or 2D materials within the same wafer, the straight and parallel edges produced by pixelation or cutting determine the twist angles. For the alignment of heterostructures or 2D materials from different growth wafer, the flat of sapphire wafer was used to determine the twist angles.

### Transfer of Oxide Thin Films and 2D/3D Integration

Figure S17 (Supporting Information) illustrates the process of isolating oxides thin films and integrating them with 2D materials. The oxide thin film was first deposited on MoS_2_ grown on sapphire by atomic layer deposition (SI ALD LL, SENTECH), forming a weak 2D/3D interface. Similar to the process of transferring 2D materials, a 1um Cu layer was deposited on the oxide, and TRT was used to exfoliate Cu/oxide stack. Afterward, the Cu/oxide was attached to a PDMS thin film, followed by etching of Cu. Additional 2D materials could be stacked on the oxide/PDMS using the method shown in Figure 1a, resulting in a 2D/oxide superlattice on PDMS. 2D/oxide superlattice with more supercells was obtained by repeating previous steps. The 2D/oxide superlattice could be released onto an arbitrary substrate in the same manner described in Figure 1a.

### Field‐Effect Transistor (FET) Fabrication and Electrical Measurements

Monolayer MoS_2_ was transferred onto a 35nm Al_2_O_3_/highly doped Si substrate using the process illustrated in Figure 1. Next, the channel areas were isolated by lithography (BA6 Gen4, Suss MA) and O_2_ dry etching (RIE 230ip, Samco). After etching, the photoresist mask layer on the surface of the channel material is removed by soaking in acetone, isopropanol and deionized water respectively. Then, lithography was used to define the source/drain contacts, followed by electron beam evaporation of 20 nm bismuth/30 nm Au and lift‐off procedure. No annealing was performed on the devices. All electrical measurements were carried out by a Keithley 4200A‐SCS semiconductor parameter analyzer in a closed‐cycle cryogenic probe station.

### Characterization

Optical images were taken by Olympus BX51M microscope. The surfaces of 2D materials and oxide thin films were characterized by AFM (Dimension Icon, Bruker). Raman and photoluminescence spectra were collected with a WITec Alpha300RAS using 532 nm laser excitation. The XRD Two theta‐scan and Phi‐scan measurements were carried out by a Bruker D8 Discover system with a Cu target. The thickness of the 200L 3R‐MoS_2_ was measured by SEM (Gemini500, Zeiss) and a stylus profiler (KLA‐Tencor P7).

SHG measurements were conducted using the reflection geometry of a confocal microscope (Alpha300RAS, WITec) equipped with a 1064 nm laser excitation source (NPI Rainbow 1064 OEM) with a pulse duration of 8.4 ps and a repetition rate of 50 MHz. The excitation beam was focused onto the sample with a spot size of ≈1 µm^2^ using a ×50 objective lens with a numerical aperture (NA) of 0.55. For polarization‐dependent SHG measurement, a half‐wave plate was used to control the excitation and detection, in combination with linear polarizers.

SAED, HAADF‐STEM, HRTEM were performed using a Thermo‐Fisher Talos F200X G2 instrument operating at 200 kV. For the SAED, the size of the selected area aperture during the diffraction experiment was ≈560 nm. EDS maps were conducted by a Thermo‐Fisher Super X. X‐ray photoelectron spectroscopy (XPS) was performed using a Thermo‐Fisher ESCALAB Xi+.

Cross‐sectional samples were prepared with a Ga+ based focused ion‐beam system (Helios G5 UX, Thermo‐Fisher). A 15 nm carbon and 15 nm platinum were first deposited on sample by thermal evaporation (EM ACE600, LEICA), followed by carbon electron beam deposition (200 nm) and platinum ion beam deposition (2 µm) to prevent damage and heating effects during focused ion‐beam milling.

## Conflict of Interest

The authors declare no conflict of interest.

## Author Contributions

W.K. supervised the project. W.K. and W.L. designed the experiment. W.L. carried out the transfer and stacking process and optical characterization. H.Z. performed the TEM measurements. Y.M. measured SEM and XRD. X.X. performed the fabrication and test of MoS_2_ FET. J.S., L.Y., and C.J. contribute to the growth of TMDCs. J.S. performed the LEED and verified the single crystallinity of the TMDCs. M.H., M.W., and K.L. conducted growth of single crystalline graphene. L.W. conducted growth of single crystalline BN. T.W. and Y.D. conducted growth of multilayer BN. L.S. and K.M. carried out laser pixelation of stacked 2D materials. H.C. performed the deposition of metal and oxides. All authors verified the manuscript.

## Supporting information



Supporting Information

Supplemental Video 1

## Data Availability

The data that support the findings of this study are available from the corresponding author upon reasonable request.
