# Supplementary material for: 3D Crystal Construction by Single‐Crystal 2D Material Supercell Multiplying
Source: Adv Sci (Weinh). 2024 Nov 18;12(2):2411656. doi: 10.1002/advs.202411656 (PMC11727270; doi:10.1002/advs.202411656)
Supplement: Supplementary file 1 — Supporting Information [file ADVS-12-2411656-s001.docx]

**Three-dimensional Crystal Construction by Single-crystal Two-dimensional Material Supercell Multiplying**

*Wenhao Li, Jichuang Shen, Yaqing Ma, Xiang Xu, Han Chen, Lida Yu, Chen Ji, Menglin He, Kezhao Ma, Yiwei Duo, Li Wang, Tongbo Wei, Liping Shi, Muhong Wu, Kaihui Liu, Huaze Zhu*, Wei Kong**

**.**

**Table of Contents:**

**Figure S1**. Single crystallinity of CVD-grown monolayer MoS_2_ 1

**Figure S2**. Photograph of a 2-inch 10L MoS_2_ wafer on PDMS. 2

**Figure S3.** Optical images and Raman spectra of transferred TMDCs from sapphire. 3

**Figure S4.** Transfer of MoS_2_ grown on SiO_2_. 4

**Figure S5.** Optical images and characterizations of transferred graphene and BN. 5

**Table S1.** Wafer-scale stacking method for the CVD-grown 2D materials 6

**Figure S6.** Compatibility of the transfer process with arbitrary substrates. 8

**Note S1.** Residue evaluation of the transfer process. 9

**Figure S7**. XPS of the transferred MoS_2_. 10

**Note S2.** Cleanliness and integrity of transferred MoS_2_. 11

**Figure S8.** Characterizations of cleanliness and integrity of transferred MoS_2_. 12

**Figure S9.** Clean interface and coupling between the transferred 2D materials. 13

**Figure S11.** Characterizations of MoS_2_ before and after transfer process. 15

**Figure S12.** MoS_2_ FET performance. 16

**Figure S13.** SHG spectra of artificial bilayer 3R-MoS_2_, artificial bilayer 2H-MoS_2_ and monolayer MoS_2_. 17

**Note S3.** Twist angle alignment method. 18

**Figure S14**. Algiment between interlayer 2D layers. 19

**Figure S15**. Precise twist angle control. 20

**Figure S16**. Characterizations of 200L 3R-MoS_2_ 21

**Note S4.** Van der Waal 2D/3D integration. 22

**Figure S17.** Schematic of the transfer process for oxide thin films and 2D/Oxide integration. 23

**Figure S18**. AFM topographies of transferred oxide thin film 24

**Figure S19.** Optical images of transferred oxides. 25

**Nore S5.** Characterizations of ALD deposited Al_2_O_3_ thin film on MoS_2_. 26

**Figure S20**. Characterizations of transferred Al_2_O_3_. 27

**Figure S21.** Photograph of a 2-inch MoS_2_/Al_2_O_3_ heterostructure on SiO_2_/Si. 28

**Note S6.** Calculation of coherence length. 29

**Note S7.** Calculation detail about SHG intensity in 2D/3D superlattice. 30

**Figure S22.** Model of coherence enhancement of SHG in MoS_2_ with inserted oxides. 31

**Figure S23.** QPM condition scaling up to three supercells. 32

**Figure S24**. Scheme of MoS_2_/oxides superlattice in Figure 4d. 33

**Note S8.** QPM condition with ML 3R-MoS_2_/oxide superlattice. 34

**Figure 25.** Optical images of Cu films after exfoliation process. 35


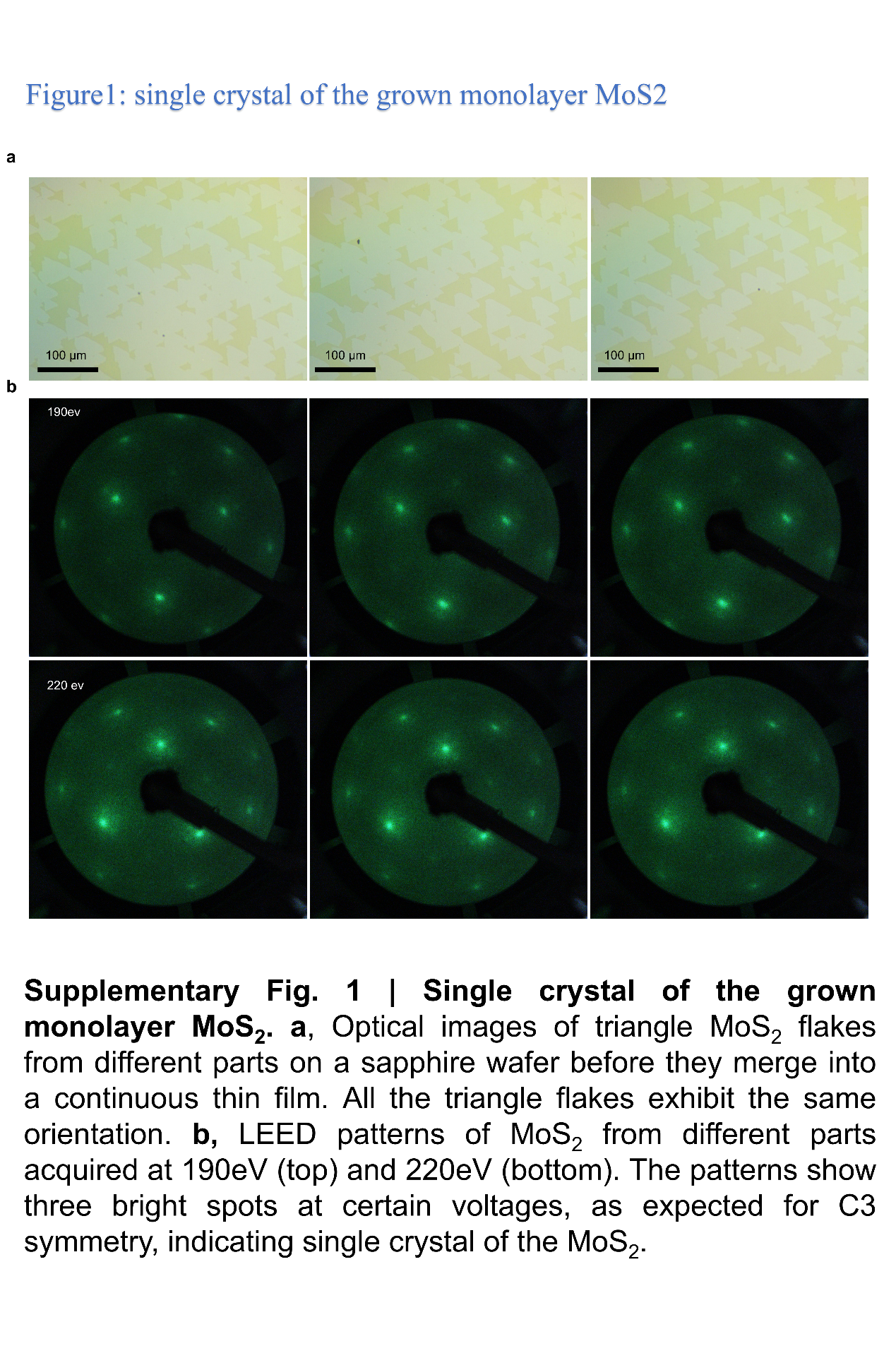


**Figure S1**. Single crystallinity of CVD-grown monolayer MoS_2_. a), Optical images of triangle MoS_2_ flakes from different parts on a sapphire wafer before they merge into a continuous thin film. All the triangle flakes exhibit the same orientation. b) Low-energy electron diffraction (LEED) patterns of MoS_2_ from different parts acquired at 190eV (top) and 220eV (bottom). The patterns show three bright spots at certain voltages, as expected for C3 symmetry, indicating single crystal of the MoS_2_.


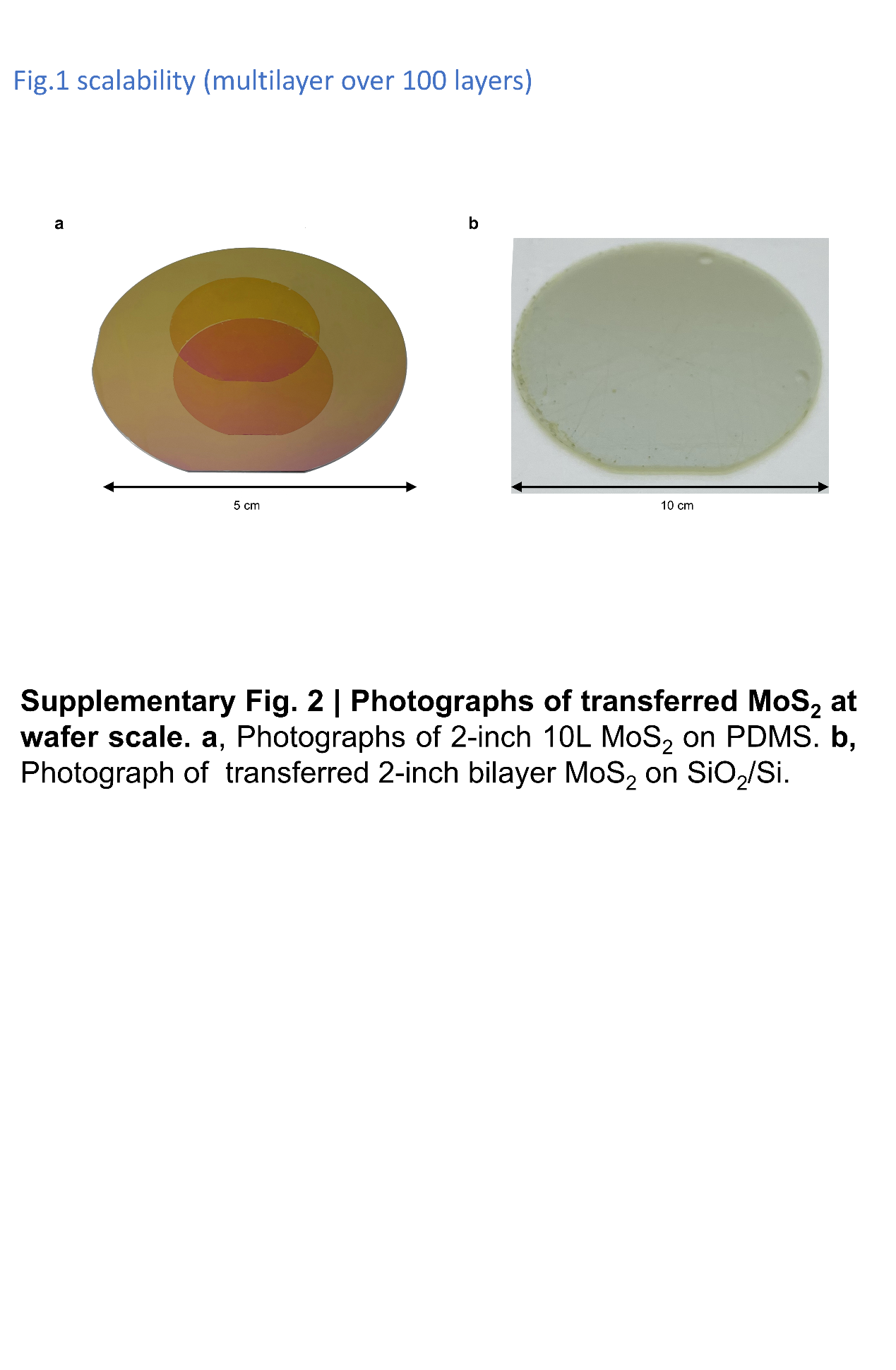


**Figure S2**. Photograph of a 2-inch 10L MoS_2_ wafer on PDMS.


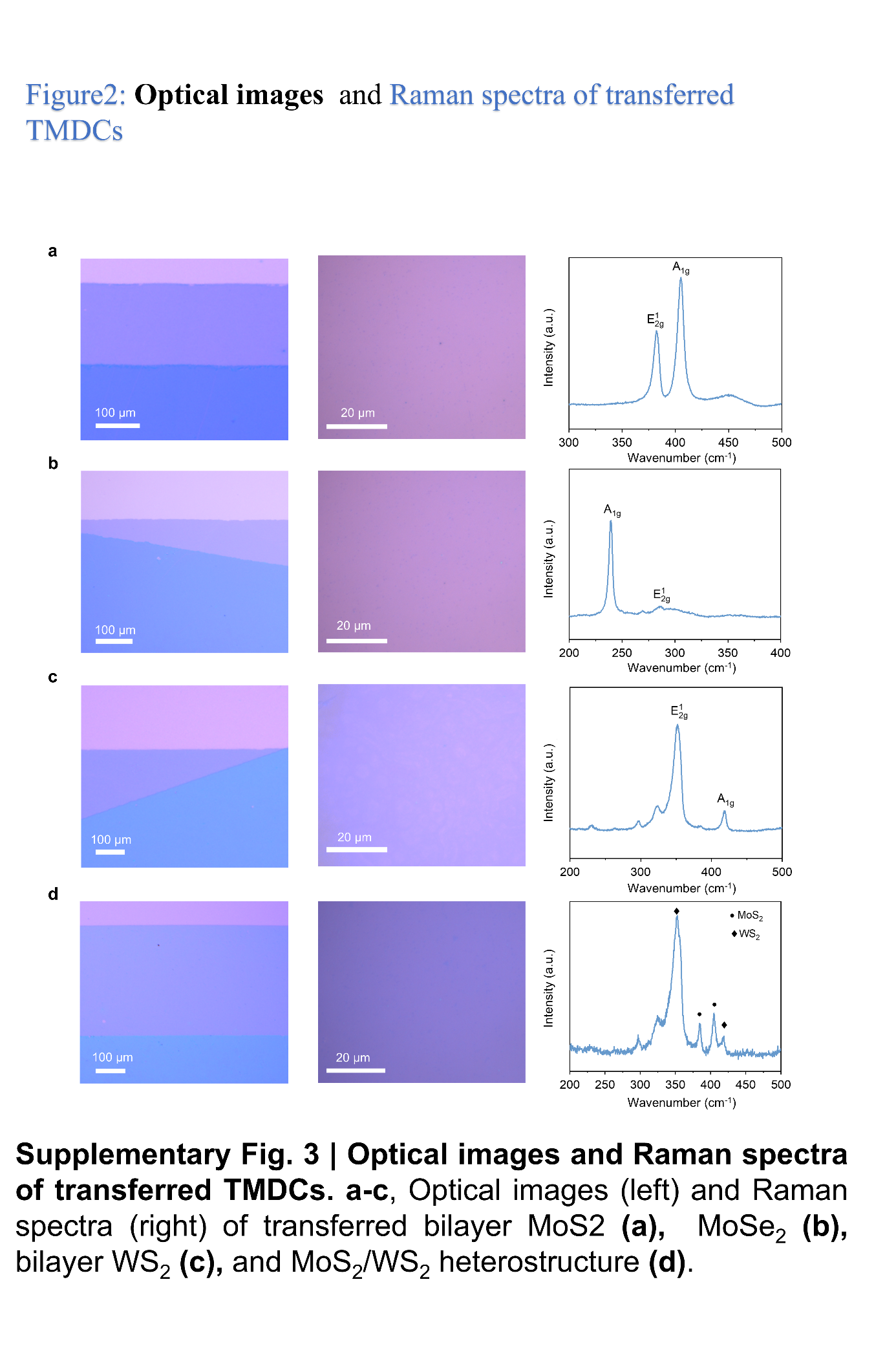


**Figure S3.** Optical images and Raman spectra of transferred TMDCs from sapphire. a-d), Optical images at ×5 (left) and ×100 (middle) magnification and Raman spectra (right) of transferred bilayer MoS_2_ (a), bilayer MoSe_2_ (b), bilayer WS_2_ (c), and MoS_2_/WS_2_ heterostructure (d).


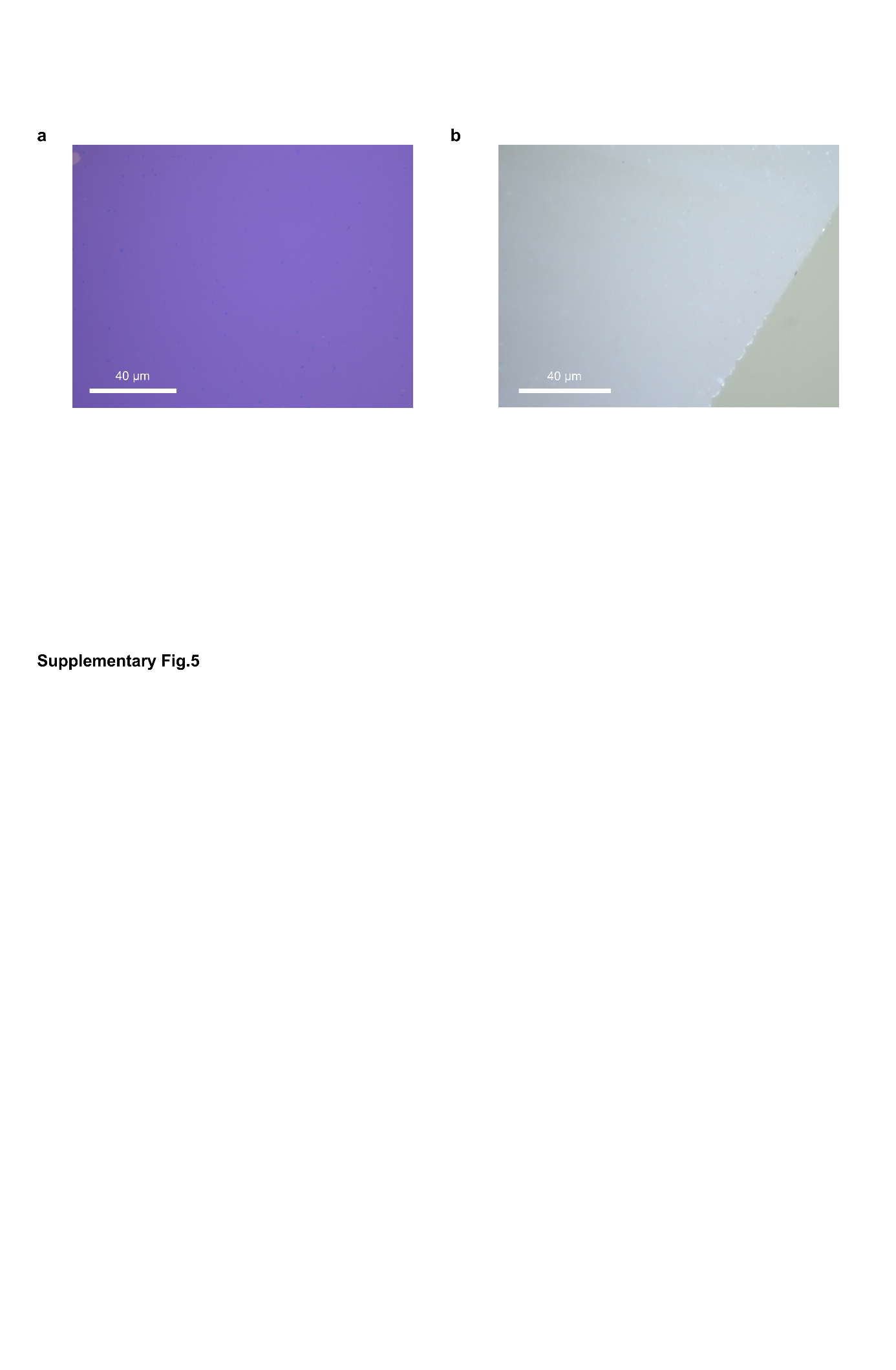


**Figure S4.** Transfer of MoS_2_ grown on SiO_2_. a), Optical image of commercial monolayer MoS_2_ grown on SiO_2_ (SixCarbon Technology Shenzheng). b), Optical image of transferred MoS₂ on glass from SiO₂. This transfer process is suitable not only for 2D materials grown on sapphire and Cu but also for those grown on SiO₂, further demonstrating the versatility of the process.


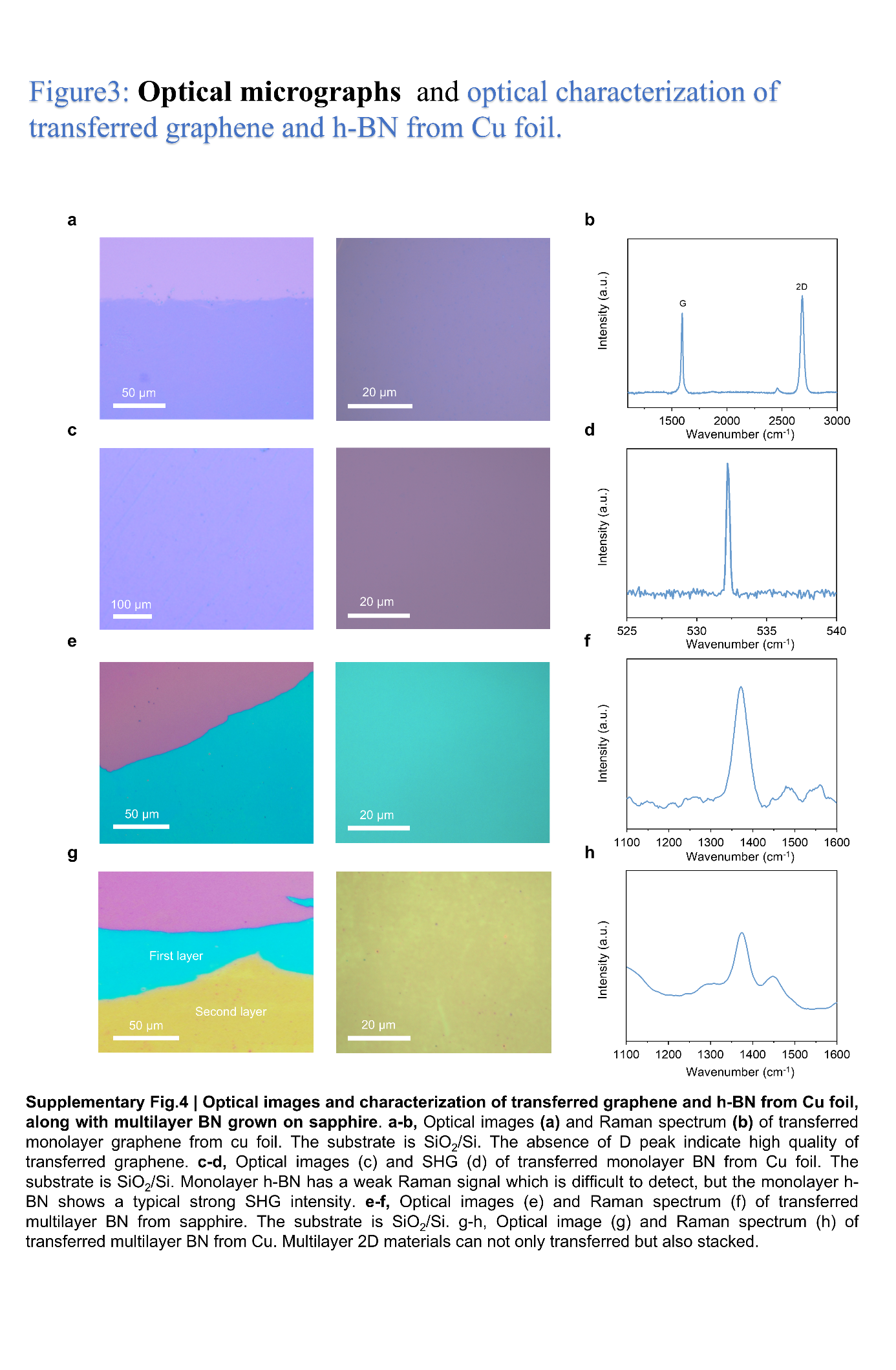


**Figure S5.** Optical images and characterizations of transferred graphene and BN. a-b), Optical images at ×5 and ×100 magnification (a) and Raman spectra (b) of transferred single-crystalline monolayer graphene from cu foil. c-d), Optical images at ×5 and ×100 magnification (c) and SHG (d) of transferred single-crystalline monolayer h-BN from Cu foil. Monolayer h-BN has a weak Raman signal which is difficult to detect, however, it shows a typical strong SHG intensity. e-f), Optical images at ×5 and ×100 magnification (e) and Raman spectrum (f) of transferred multilayer BN from sapphire. Synthesis method of multilayer BN was discussed in previous work.^[1]^ g-h), Optical images at ×5 and ×100 magnification (g) and Raman spectrum (h) of stacked thick BN from a commercial multilayer BN grown on Cu foil (SixCarbon Technology Shenzheng)

# **Table S1.** Wafer-scale stacking method for the CVD-grown 2D materials

| Type | Maximum layer number | Material | Thickness | Crystallinity | Alignment ability | Ref. |
| --- | --- | --- | --- | --- | --- | --- |
| Wet process | 3 | CVD TMDCs | Monolayer or multilayer | Poly | No | ^[2-3]^ |
| Wet process | 3 | CVD MoS_2_ | Monolayer | Poly | Yes | ^[4]^ |
| Wet process | 4 | CVD graphene | Monolayer | Poly | No | ^[5]^ |
| Wet process | 4 | CVD graphene | Monolayer | Poly | No | ^[6]^ |
| Semi-dry | 5 | CVD TMDCs, h-BN | Monolayer or multilayer | Poly | No | ^[7]^ |
| Dry process | 9 | CVD TMDCs | Monolayer | Poly | No | ^[8]^ |
| Dry process | 10 | CVD TMDCs, graphene, and BN | Monolayer or multilayer | Poly | No | ^[9]^ |
| Dry process | 80 | CVD TMDCs | Monolayer | Poly | Yes | ^[10]^ |
| **Semi-dry** | **200** | **CVD TMDCs, graphene, BN, and oxides** | **Monolayer or multilayer** | **Poly or Single** | **Yes** | **This work** |

The wafer-scale stacking methods for CVD-grown 2D materials, as illustrated in Supplementary Table 1, can be classified into three categories: wet, semi-dry, and dry processes. The wet process is versatile when paired with an appropriate etchant, but it has significant drawbacks, including uncontrollable angular alignment, time-consuming procedures due to the floating process, and a high risk of contamination from polymer supports. The dry transfer method uses 2D-2D adhesion to overcome 2D-substrate adhesion, isolating the 2D materials. However, this method is not suitable for 2D materials with strong 2D-substrate adhesion, such as single-crystalline graphene and BN grown on metal substrates.^[11]^ Consequently, its application is restricted to polycrystalline 2D materials grown on substrates like SiO_2_/Si or sapphire. Thus, stacking of single- crystalline 2D materials based on CVD samples was usually constrained in micro-sized domains.^[12-13]^ While dry transfer allows for control of the twist angle, the in-plane order of stacking is still limited by the polycrystalline nature of the materials used. The metal-assisted semi-dry process offers versatility in the selection of 2D materials, but previous implementations have been confined to layer-by-layer transfer, making it challenging to scale vertically.

In contrast, our method significantly broadens the range of applicable building blocks and is compatible with a wider variety of 2D materials, including, but not limited to, TMDCs, graphene, and h-BN. This method is effective for both monolayer and multilayer structures, encompassing both single-crystalline and polycrystalline 2D materials, and it can also be applied to oxides of varying thicknesses. Additionally, it offers precise control over twist angles and enables high stacking efficiency through multilayer-by-multilayer stacking. By avoiding the uncontrollable floating process, our method is easily automated, making it well-suited for integration with standard mass production processes commonly used in the industry, such as the mass-transferring process for micro-light-emitting diodes fabrication via stamping^[14-15]^. This advancement paves the way for the creation of macroscopic complex artificial crystals and the exploration of their novel functionalities and high-performance applications.


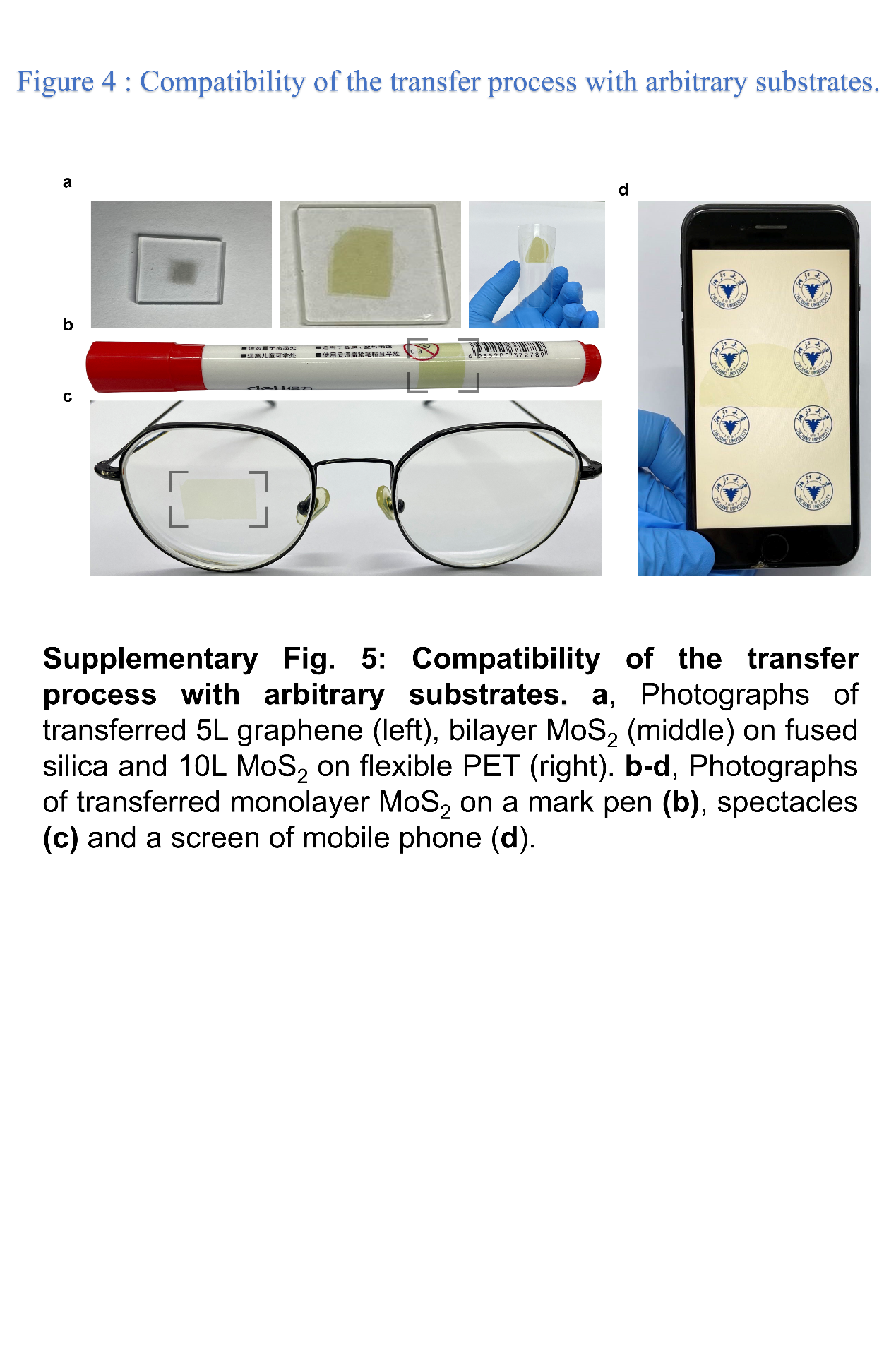


**Figure S6.** Compatibility of the transfer process with arbitrary substrates. a), Photographs of transferred 5L graphene (left), bilayer MoS_2_ (middle) on fused silica and 10L MoS_2_ on flexible polyethylene terephthalate (PET) (right). b-d), Photographs of transferred monolayer MoS_2_ on a mark pen (b), spectacles (c) and a screen of mobile phone (d).

# **Note S1.** Residue evaluation of the transfer process.

Compared to the conventional PDMS transfer method, our process leaves minimal polymer residues. In the traditional PDMS process, 2D materials are transferred to the target substrate layer by layer, with PDMS coming into contact with the surface of each layer.^[4, 16]^ This repeated contact results in polymer residues being left on each layer's surface, which hinders the formation of a clean 2D-2D interface — crucial for preserving the material's properties. This residue hinders the formation of a clean 2D-2D interface, which is crucial for maintaining material properties. In contrast, our process ensures that only the uppermost layer makes contact with PDMS, thereby maintaining a clean 2D-2D interface.

We used X-ray photoelectron spectroscopy (XPS) to evaluate the residue content, as shown in Figure S7a-f. The residue amount was determined by analyzing the content of carbon-oxygen bonds, such as C-O and C=O, among all carbon bonds. We prepared 2D stacks with varying numbers of layers using both our process and the conventional PDMS method. For comparison, we also included data from as-grown MoS₂ on sapphire and MoS₂ transferred using the conventional wet process with polymethyl methacrylate (PMMA) (see Figure S7g-h).

The results (Figure S7i) indicate that the wet process leaves the most residue. Our process results in significantly less residue than the conventional PDMS method, and the difference becomes more pronounced with an increasing number of layers, as residue from the conventional PDMS process accumulates with each additional layer. Moreover, the clean surface on the side that does not contact PDMS was confirmed by AFM topography, showing an RMS of 0.56 nm, as depicted in Figure S8.


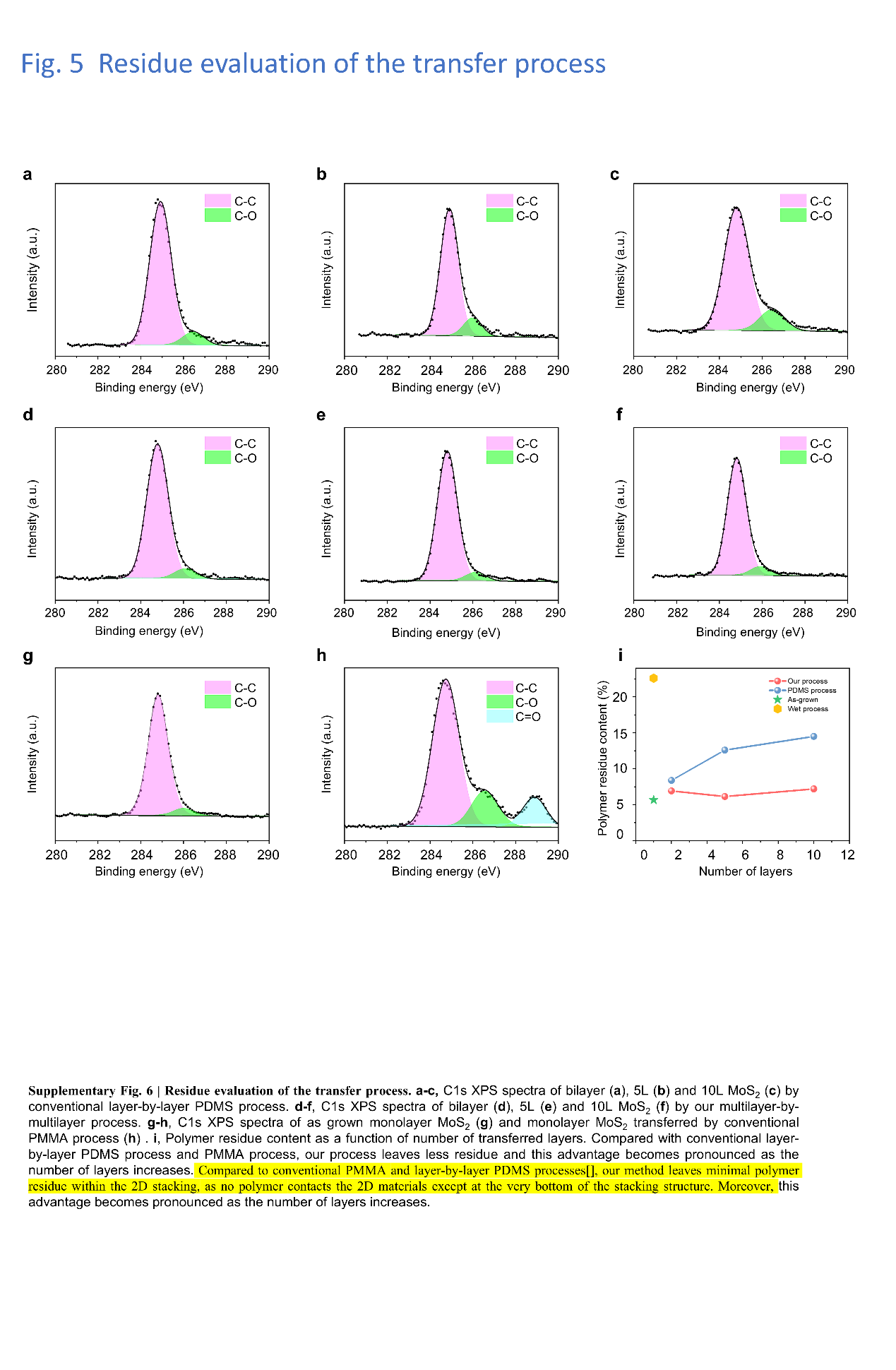


**Figure S7**. XPS of the transferred MoS_2_. a-c) C1s XPS spectra of bilayer (a), 5L (b) and 10L MoS_2_ (c) by conventional layer-by-layer PDMS process. d-f) C1s XPS spectra of bilayer (d), 5L (e) and 10L MoS_2_ (f) by our supercell multiplying process. g-h) C1s XPS spectra of as-grown monolayer MoS_2_ (g) and monolayer MoS_2_ transferred by conventional wet process (h). i) Polymer residue content as a function of number of transferred layers. Compared with conventional layer-by-layer PDMS process and wet process, our process leaves minimal residue and this advantage becomes more pronounced as the number of layers increases.

# **Note S2.** Cleanliness and integrity of transferred MoS_2_.

The transfer process for 2D materials can be divided into two primary stages: (1) exfoliation of MoS₂ from the growth substrate onto PDMS, and (2) release from PDMS onto an SiO₂/Si substrate. To assess the cleanliness and structural integrity of the transferred MoS₂ over a large area, AFM measurements were conducted at nine locations across the entire 2-inch MoS₂ wafer (see Figure S8a). In the first stage, AFM topography of the MoS₂ on PDMS displayed a clean, residue-free surface, with no detectable contamination from Cu or its etchant (see Figure S8b). The surface roughness across the 2-inch wafer ranged from 0.4 to 0.7 nm, with a Gaussian distribution, confirming a uniformly clean surface (see Figure S8c).

In the second stage, after transfer onto the SiO₂/Si substrate, most areas of the MoS₂ wafer remained clean, while very few localized regions exhibited minor wrinkles, bubbles, and residues, causing slight variations in roughness (see Figure S8d-f). Based on AFM data, the density of wrinkles and bubbles (or residues) on the transferred 2-inch MoS₂ wafer on SiO₂/Si was estimated at approximately 0.02 μm/μm² and 0.3% (area ratio) respectively.


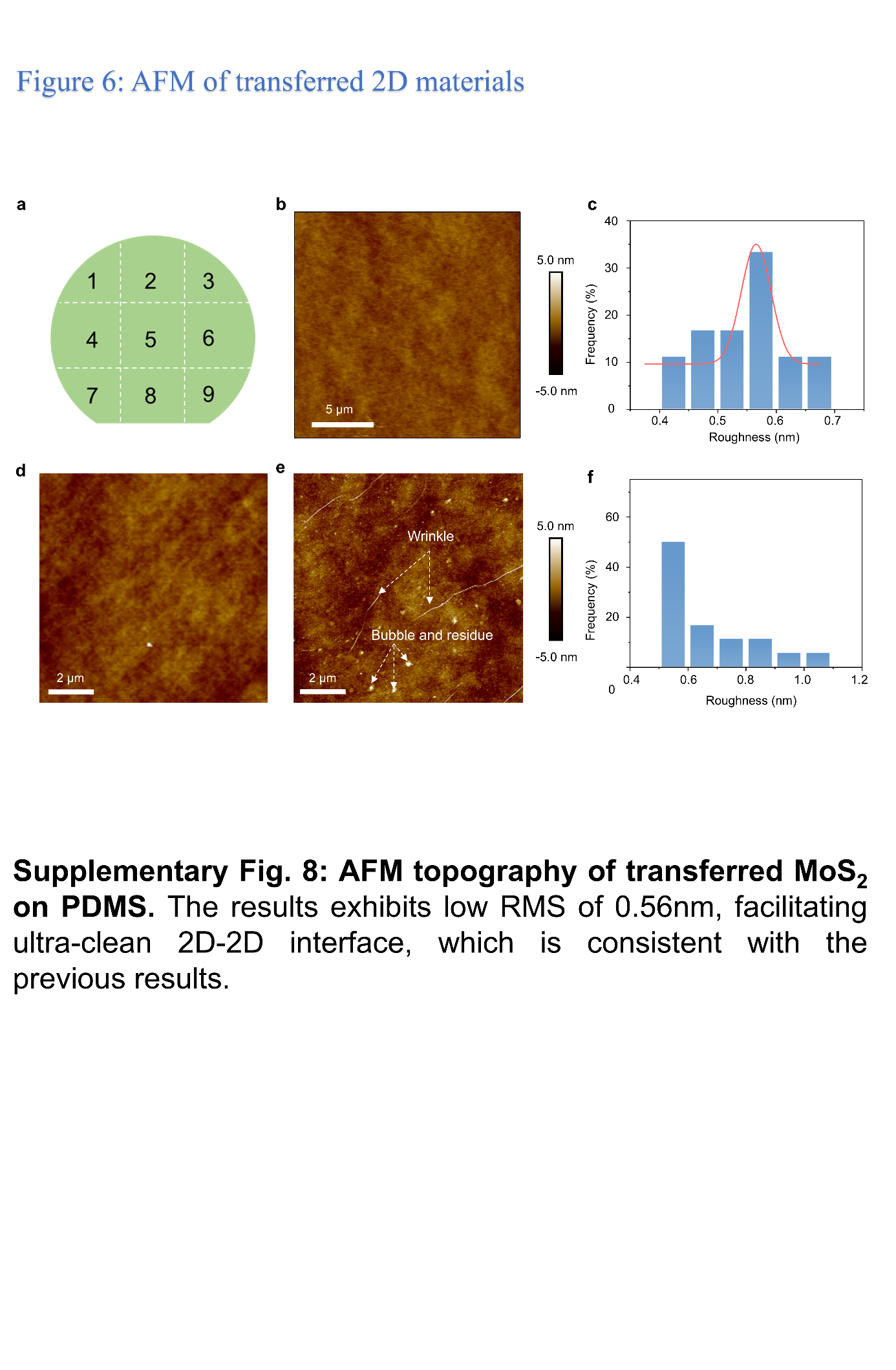


**Figure S8.** Characterizations of cleanliness and integrity of transferred MoS_2_. a), Locations of AFM test. Nine test sections are divided across the entire 2-inch wafer and eighteen AFM measurements are conducted. b), Representative AFM topography of transferred MoS_2_ on PDMS (after etching of Cu and before release to SiO_2_/Si). c), Roughness distribution of 2-inch MoS_2_ wafer on PDMS. The line is the Gaussian fitting curve. d-e), representative AFM topography of a clean area (d) and the localized area with wrinkles, bubbles and residue (e). f), Roughness distribution on a transferred 2-inch MoS_2_ wafer on SiO_2_/Si.


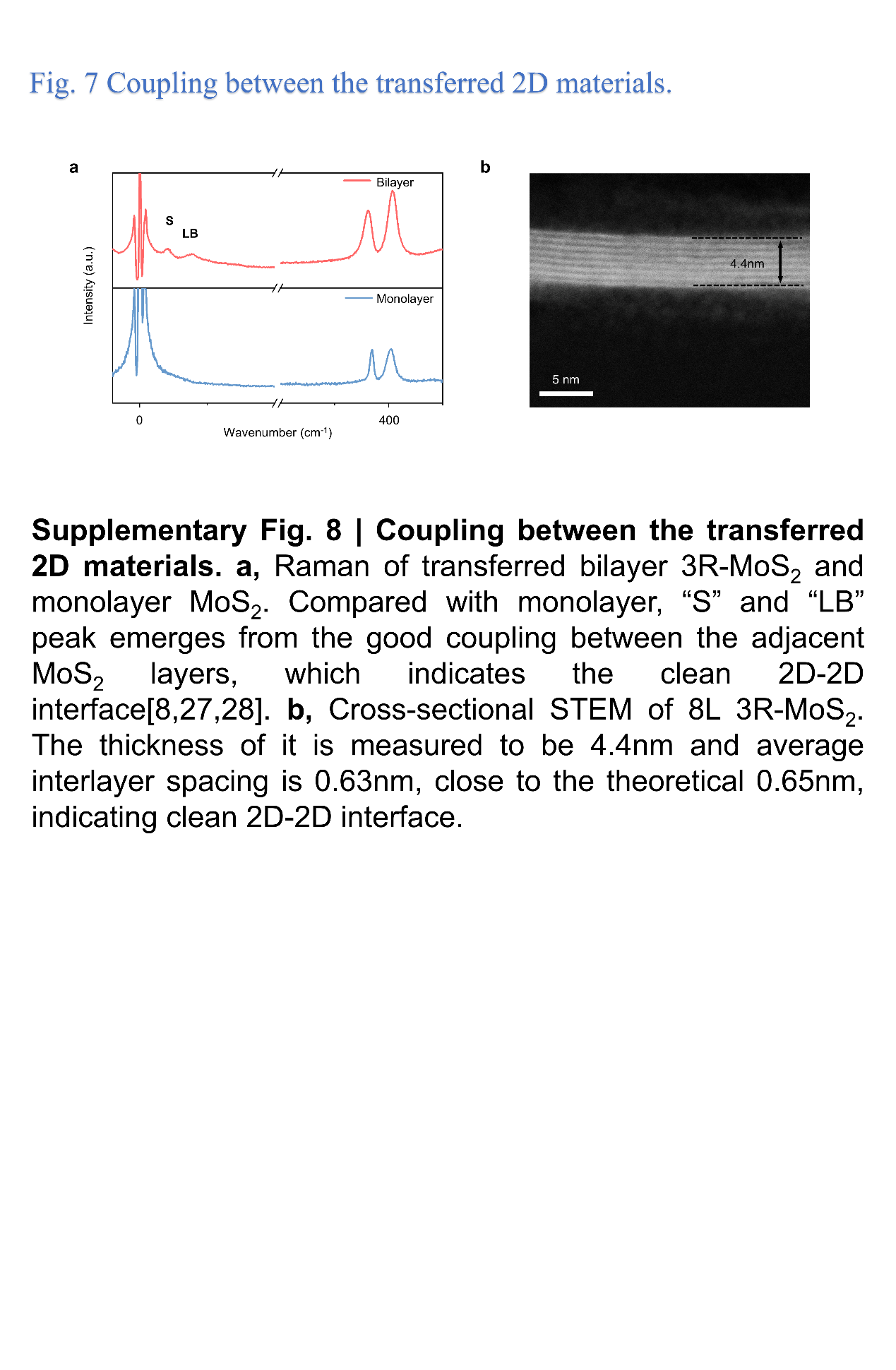


**Figure S9.** Clean interface and coupling between the transferred 2D materials. a) Raman spectra of transferred bilayer 3R-MoS₂ and monolayer MoS₂. Compared with the monolayer, the “S” and “LB” peaks emerge due to the strong coupling between adjacent MoS₂ layers.^[17-19]^ b) Cross-sectional HAADF–STEM image of 8-layer 3R-MoS₂. The measured thickness is 4.4 nm, and the average interlayer spacing is 0.63 nm, which is close to the theoretical value of 0.65 nm. This indicates that there is no contamination trapped between the 2D materials and that a clean 2D–2D interface has been achieved.


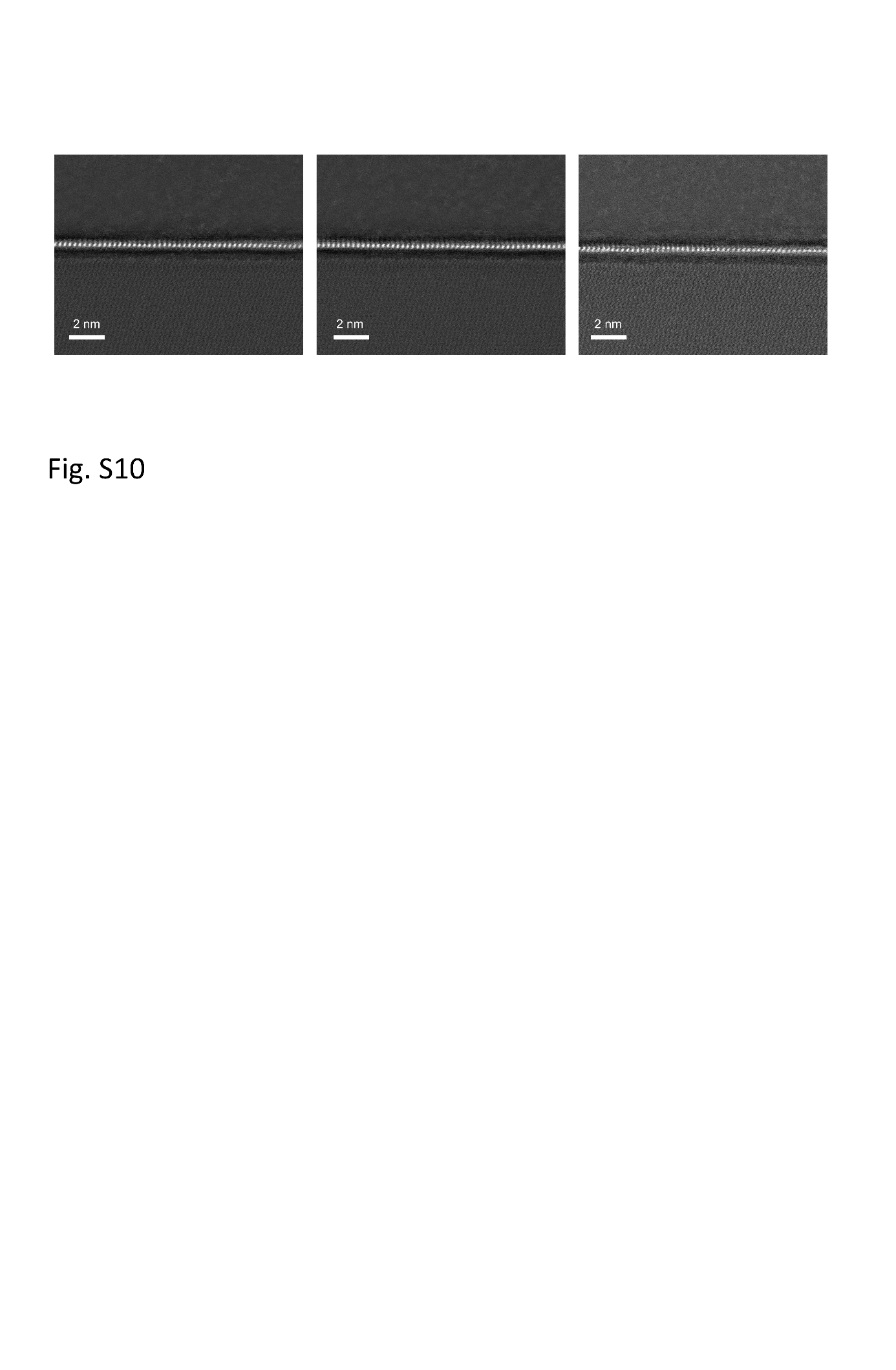


Figure S10. Cross-sectional HADDF-STEM images of MoS_2_ on sapphire from different locations after sequential Cu deposition and etching processes. Clear atomic images confirm that the deposition and etching processes do not damage the 2D materials.


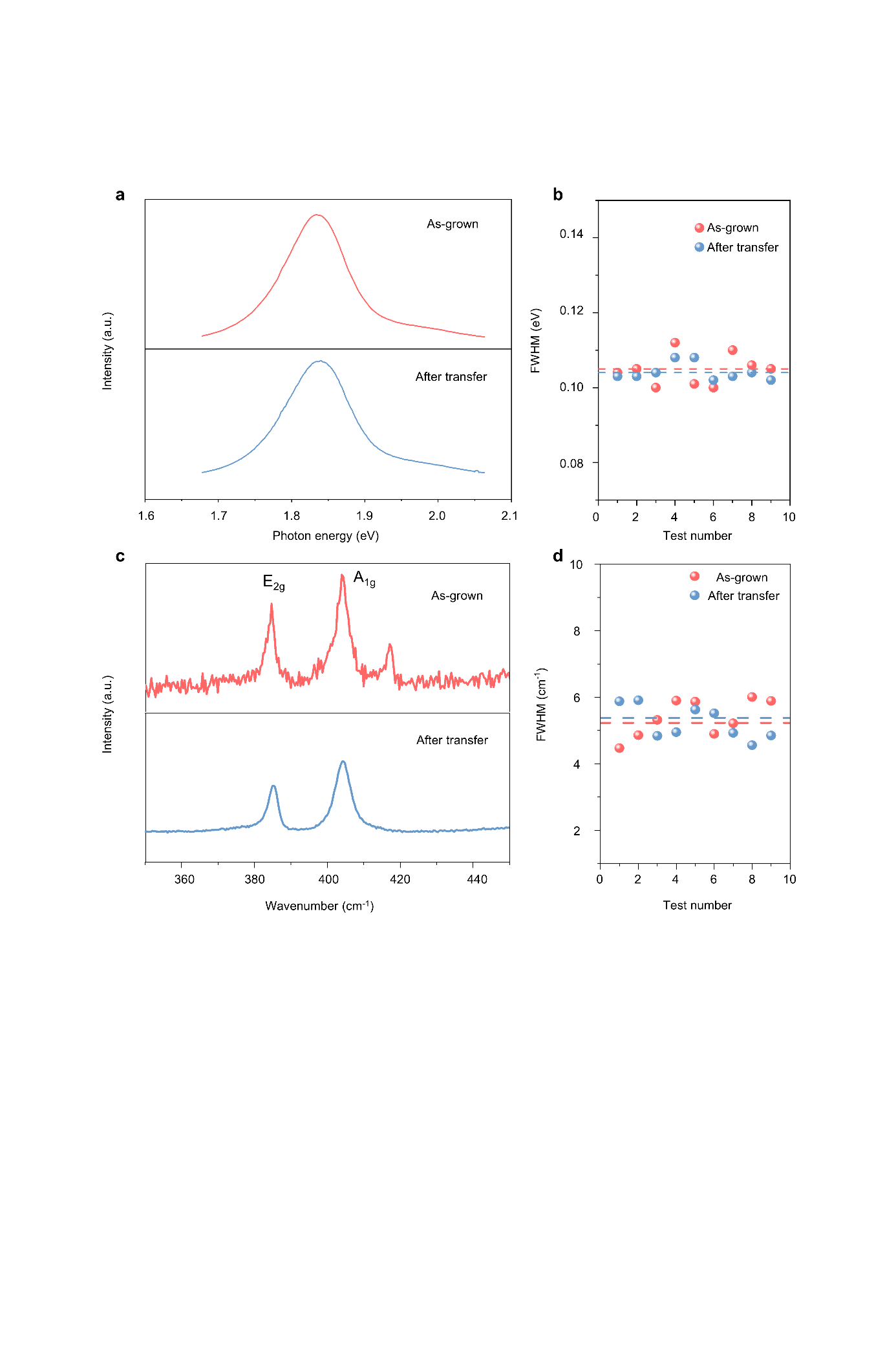


**Figure S11.** Characterizations of MoS_2_ before and after transfer process. a), Representative Photoluminescence spectra of monolayer MoS_2_. b), Full width at half Maxima (FWHMs) of Photoluminescence from different locations in a 2-inch wafer. Dashed lines are average FWHMs. c) Raman spectra of monolayer MoS_2_. d), FWHMs of A_1g_ peak in Raman spectra from different locations in a 2-inch wafer. Dashed lines are average FWHMs. No significant change was observed in either Photoluminescence or Raman spectra after transfer, indicating no deterioration in transfer process (the additional Raman peak at around 417cm^-1^ of as-grown MoS_2_ emerges from the sapphire substrate). FWHMs of both Raman and Photoluminescence spectra from different locations remains almost unchanged after transfer, indicating few defects are induced across the entire 2-inch area during the transfer process. The E_2g_-A_1g_ separation of as-grown and transferred MoS_2_ is typical 19 cm⁻¹, indicating no significant intrinsic strain in the MoS₂.


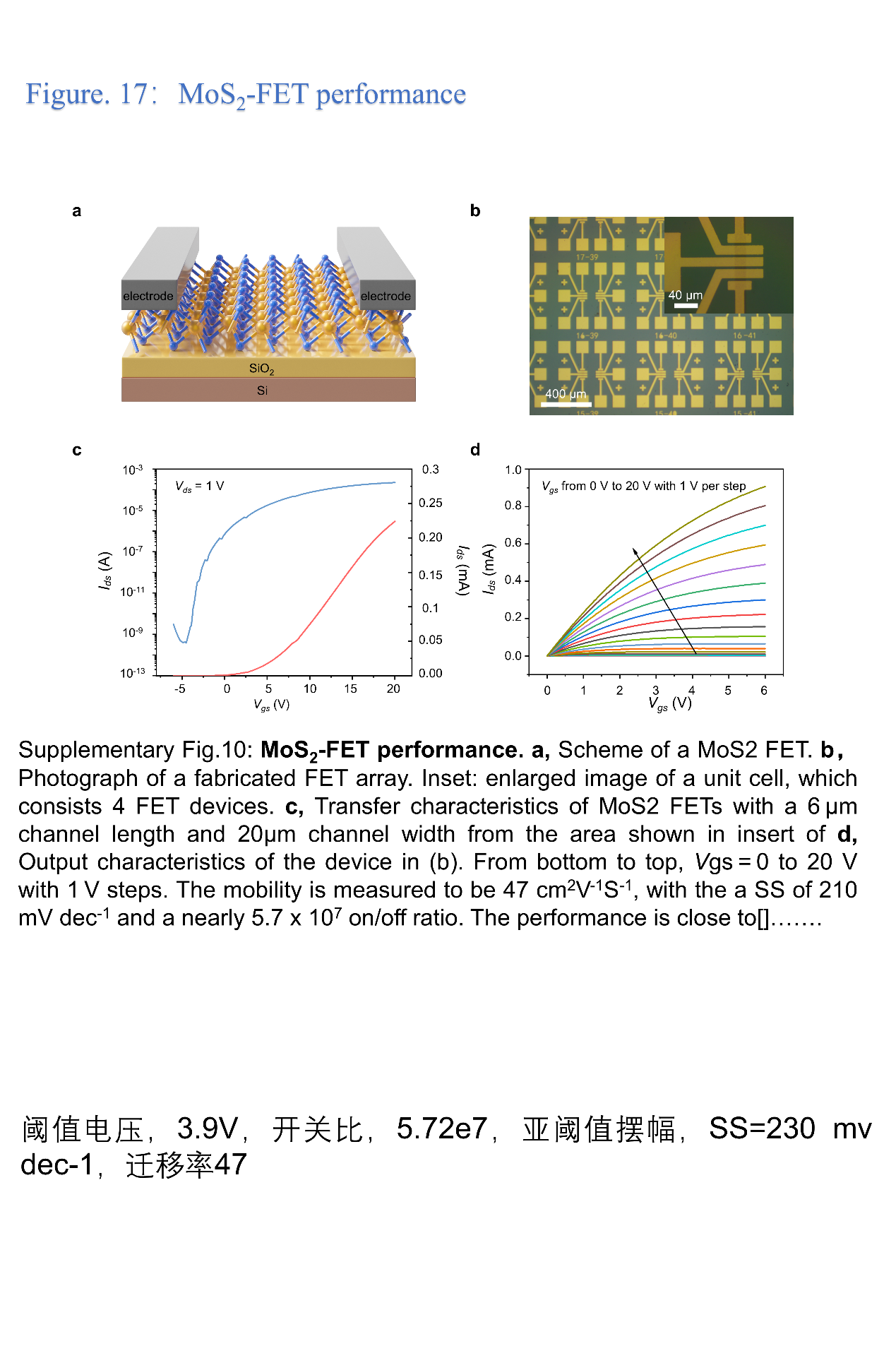


**Figure S12.** MoS_2_ FET performance. a) Scheme of a MoS_2_ FET. b) Photograph of a fabricated FET array. Inset: enlarged image of a unit cell, which consists of four FET devices. c) Transfer characteristics of MoS_2_ FETs with a 6 μm channel length and 20μm channel width from the area shown in insert of (b). d) Output characteristics of the device in (b). From bottom to top, *V*_gs_ = 0 to 20 V with 1 V steps. The mobility is measured to be 47 cm^2^V^-1^s^-1^, with a subthreshold swing of 210 mV dec^-1^ and a nearly 5.7 x 10^7^ on/off ratio. The performance is comparable to the typical MoS_2_ FETs reported in literatures,^[20-22]^ proving the reliability of the transfer process.


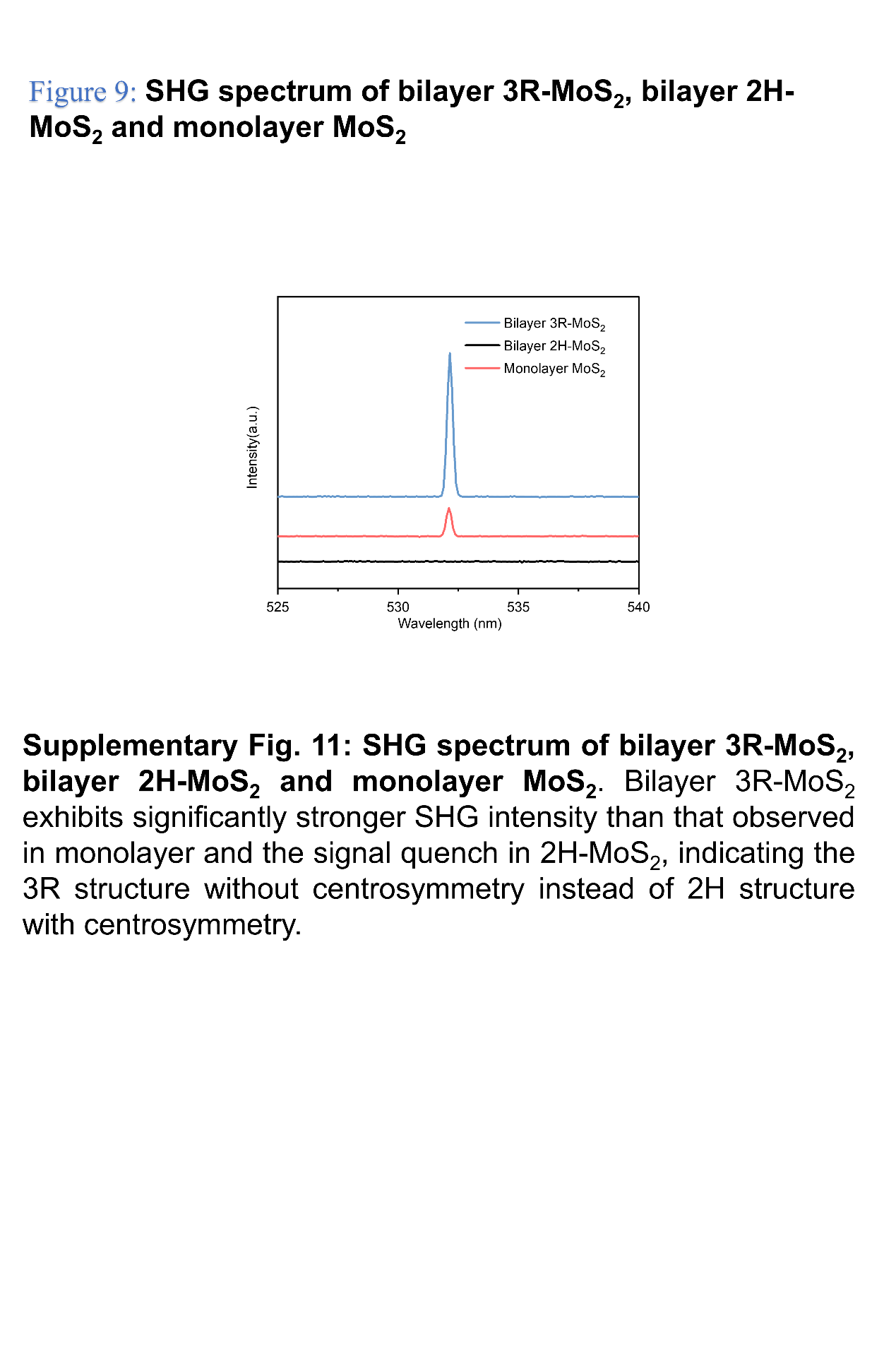


**Figure S13.** SHG spectra of artificial bilayer 3R-MoS_2_, artificial bilayer 2H-MoS_2_ and monolayer MoS_2_. The Artificial bilayer 3R-MoS_2_ exhibits significantly stronger SHG intensity than that observed in monolayer, showing centrosymmetry breaking. The SHG signal is quenched in artificial bilayer 2H-MoS_2_, indicating centrosymmetric structure.

# **Note S3.** Twist angle alignment method.

In this work, aligment between interlayer 2D layers was achieved using wafer flat (or straight edges fromed in pixelation) under a microscope with a CCD camera, as shown in Figure S14a. Three main factors may contribute to alignment errors. The first error, δ1, arises from the misorientation between MoS_2_ and sapphire. Since the growth of single-crysttline MoS_2_ on saphire with a strict epitaxial relationship, in which ($10\bar{1}$0) plane of MoS_2_ is precisely parallel to the ($11\bar{2}$0) plane of sapphire, δ1 can be considered negligible. Second error, δ2, originates from the commercial sapphire’s flat, which is ± 0.1°. The third error, δ3, comes from aligment operation under the microscope, determined by the resolution of CCD camera and line width of the flat in the optical images. The resolution of CCD camera used is 1440 × 1080 pixels, and the line width of the flat in optical images is around 3-4 pixels (see Figure S14b). A relative rotation of 0.2° between the two wafers would cause a position shit of approximately 5 pixels (1400 × sin(0.2°) ≈ 5), which is comparable to the width of the flat, making a 0.2° shift detectable. Therefore, the total algiment error, δ = δ1 + δ2 + δ3, is 0.3°, which could be further enhanced by using a higher resolution camera. Figure S14c-d shows bilayer MoS_2_ of 6° and 10° aligned usding this method. Their twist angles was further comfired by the SAED and Polarization-dependent SHG (See Figure S15a-b).


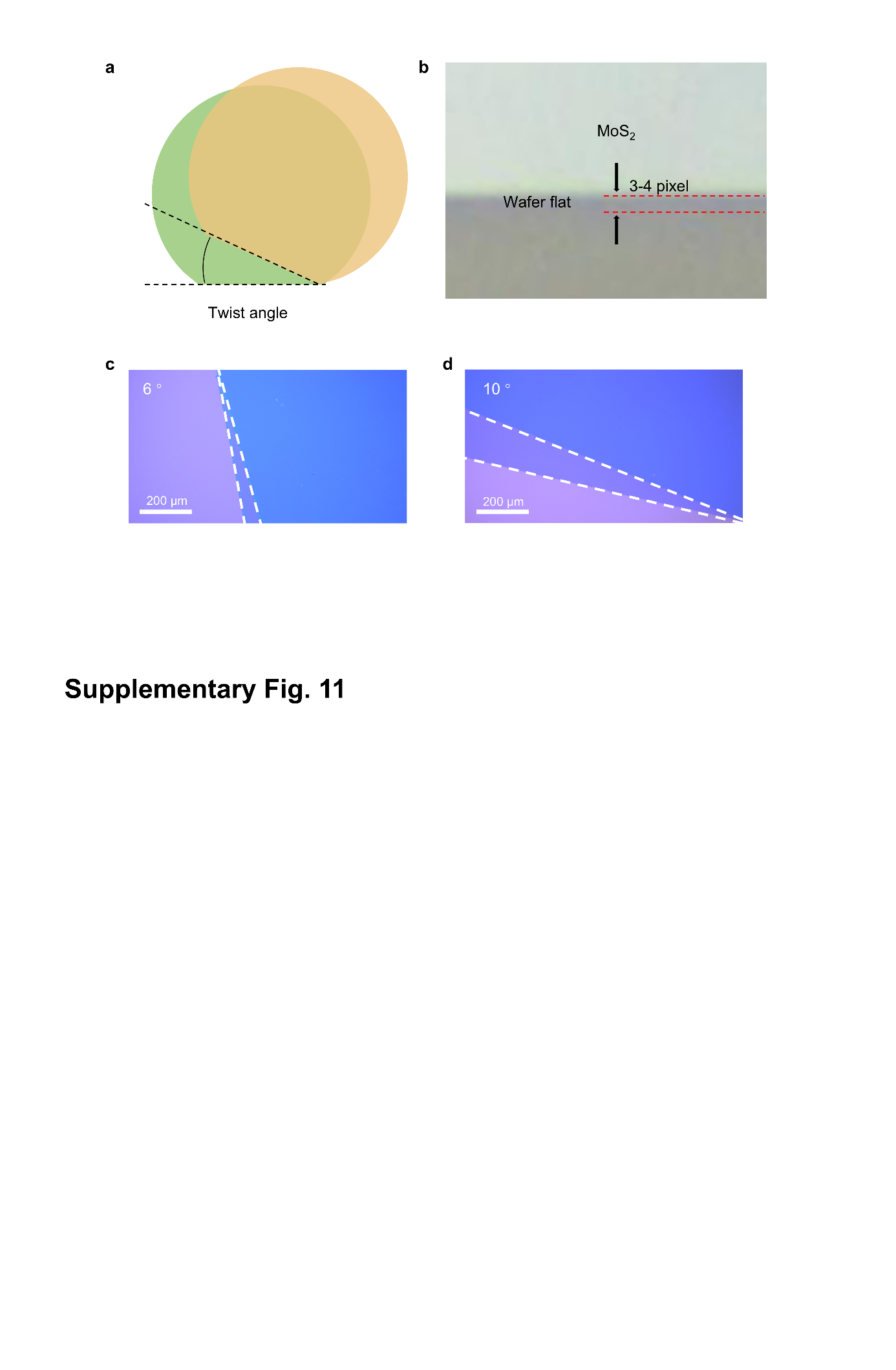


**Figure S14**. Algiment between interlayer 2D layers. a) Scheme for twsit angle determined by wafer flats. b) Width of wafer flat under the CCD camera. c-d) Optical images of bilayer MoS_2_ with the twist angles of 6° (c) and 10° (d).


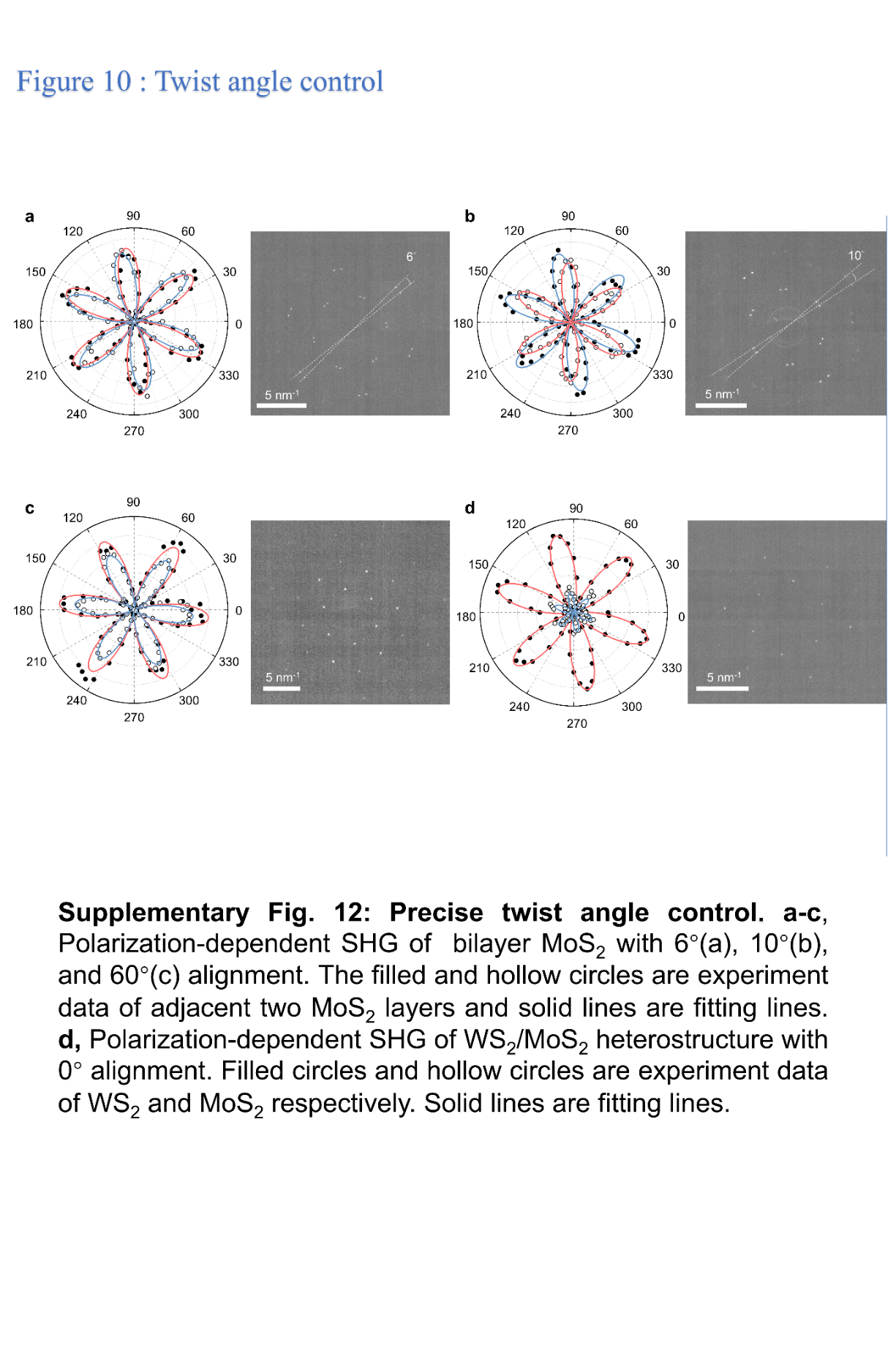


**Figure S15**. Precise twist angle control. a-c) Polarization-dependent SHG (left) and SAED (right) of bilayer MoS₂ with 6° (a), 10° (b), and 60° (c) alignment. The filled and hollow circles represent experimental data for the two adjacent MoS₂ layers, while the solid lines are the corresponding fitting curves. d) Polarization-dependent SHG (left) and SAED (right) of a WS₂/MoS₂ heterostructure with 0° alignment. The filled circles represent experimental data for WS₂, and the hollow circles for MoS₂, with solid lines indicating the fitting curves. The results of SAED and polarization-dependent SHG are highly consistent.


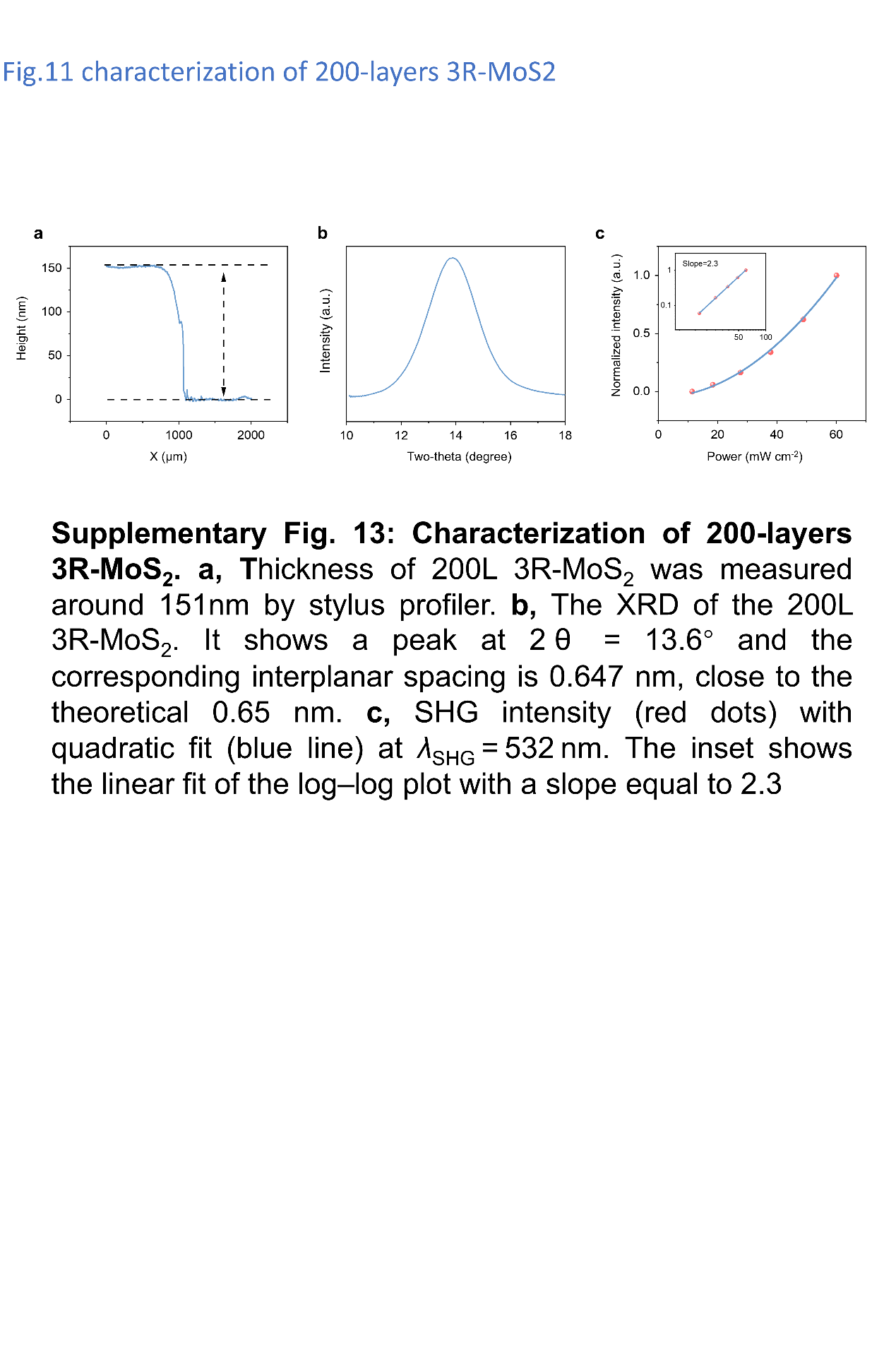


**Figure S16**. Characterizations of 200L 3R-MoS_2_**.** a) The thickness of the 200-layer 3R-MoS₂ was measured to be approximately 151 nm using a stylus profiler, consistent with the result shown in Figure 2e. b) XRD two-theta scan of the 200-layer 3R-MoS₂ shows a peak at 13.6°, corresponding to an interplanar spacing of 0.647 nm, which is close to the theoretical value of 0.65 nm. c) SHG intensity (red filled circles) with a quadratic fit (blue line) at λ_SHG_= 532 nm. The inset shows the linear fit of the log–log plot, with a slope of 2.3, close to the theoretical value of 2.^[23-24]^

# **Note S4.** Van der Waal 2D/3D integration.

Significant efforts have been made to achieve van der Waals integration between 2D and 3D materials. For example, high-κ perovskites have been incorporated into van der Waals devices to enhance electrical performance.^[25-26]^ However, this approach has been limited by the use of micrometer-sized flakes with unpredictable thicknesses. While a weak 2D-3D interface has proven effective in isolating oxide thin films, previous work has been confined to micrometer-scale areas.^[27]^ Recently, wafer-scale isolation of Al₂O₃ and its integration with 2D materials were achieved.^[28]^ In this method, an Al₂O₃ thin film was initially deposited on a sacrificial polyvinyl alcohol layer, which was later dissolved to isolate the film.

In contrast, our method deposits the oxide thin film directly onto MoS₂-covered sapphire. The weak Al₂O₃/MoS₂ interface facilitates wafer-scale isolation without the need for a sacrificial layer. This approach eliminates any contact between the oxide surface and polymers or solutions, ensuring a clean 2D/3D interface. Additionally, the absence of a floating process simplifies alignment, enhancing the angular precision.


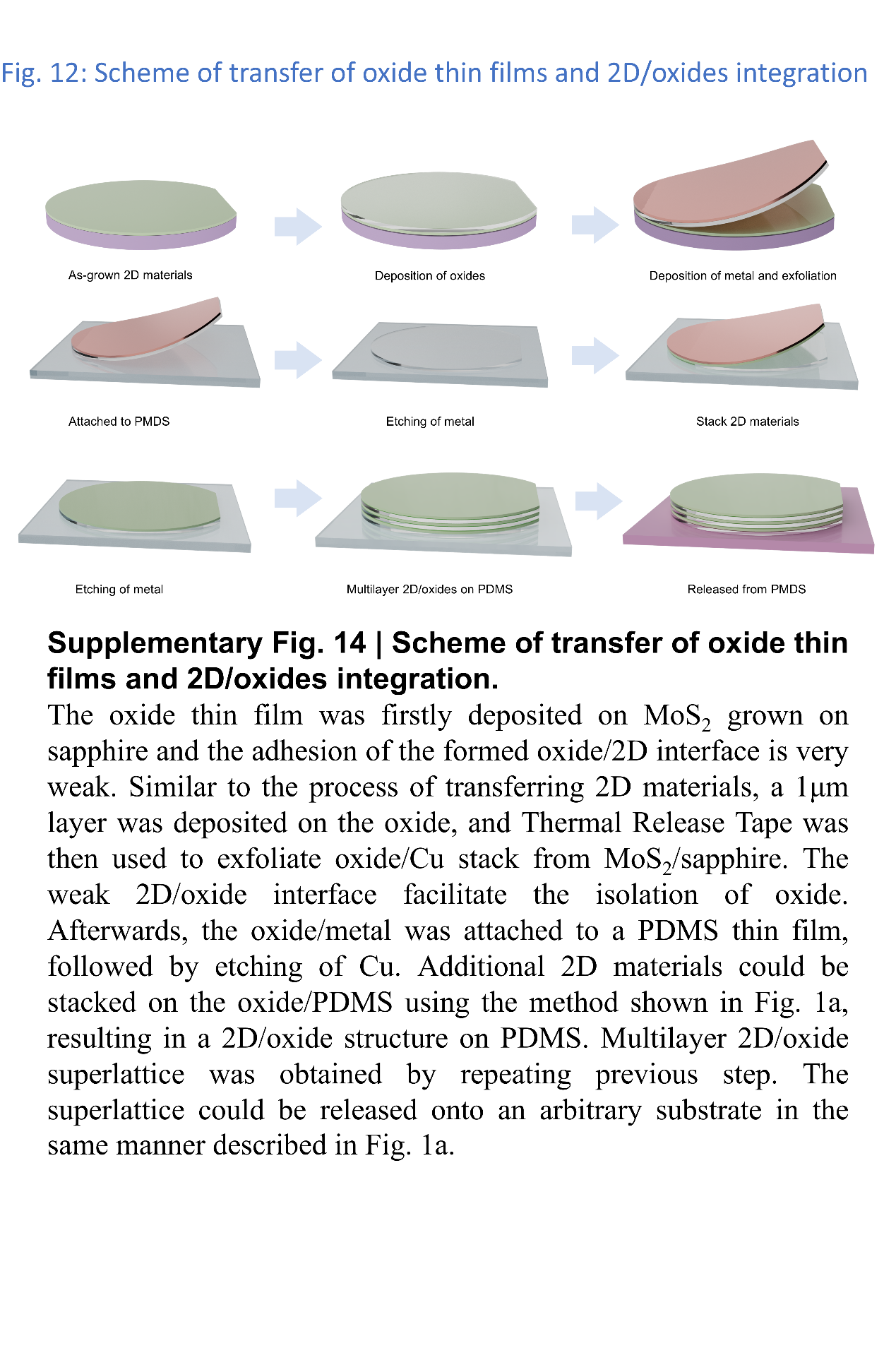


**Figure S17.** Schematic of the transfer process for oxide thin films and 2D/Oxide integration. The oxide thin film is first deposited onto MoS₂ grown on sapphire. Similar to the process used for transferring 2D materials, a 1 μm Cu metal layer is deposited on the oxide. Thermal release tape (TRT) is then used to exfoliate the Cu/oxide from the MoS₂ surface, facilitated by the weak MoS₂/oxide interface. Due to the absence of dangling bonds on 2D surfaces, the adhesion energy of 2D-oxide interfaces is generally weaker than that of 3D-3D interfaces, such as metal-oxide interfaces. The Cu/oxide layer is subsequently attached to a PDMS thin film, followed by the etching of the Cu layer. Additional monolayer or multilayer 2D materials can then be stacked onto the oxide/PDMS using the method shown in Figure 1a, resulting in a 2D/oxide heterostructure on PDMS. A 2D/oxide superlattice is obtained by repeating this step. The superlattice can be released onto an arbitrary substrate in the same manner as described in Figure 1a.


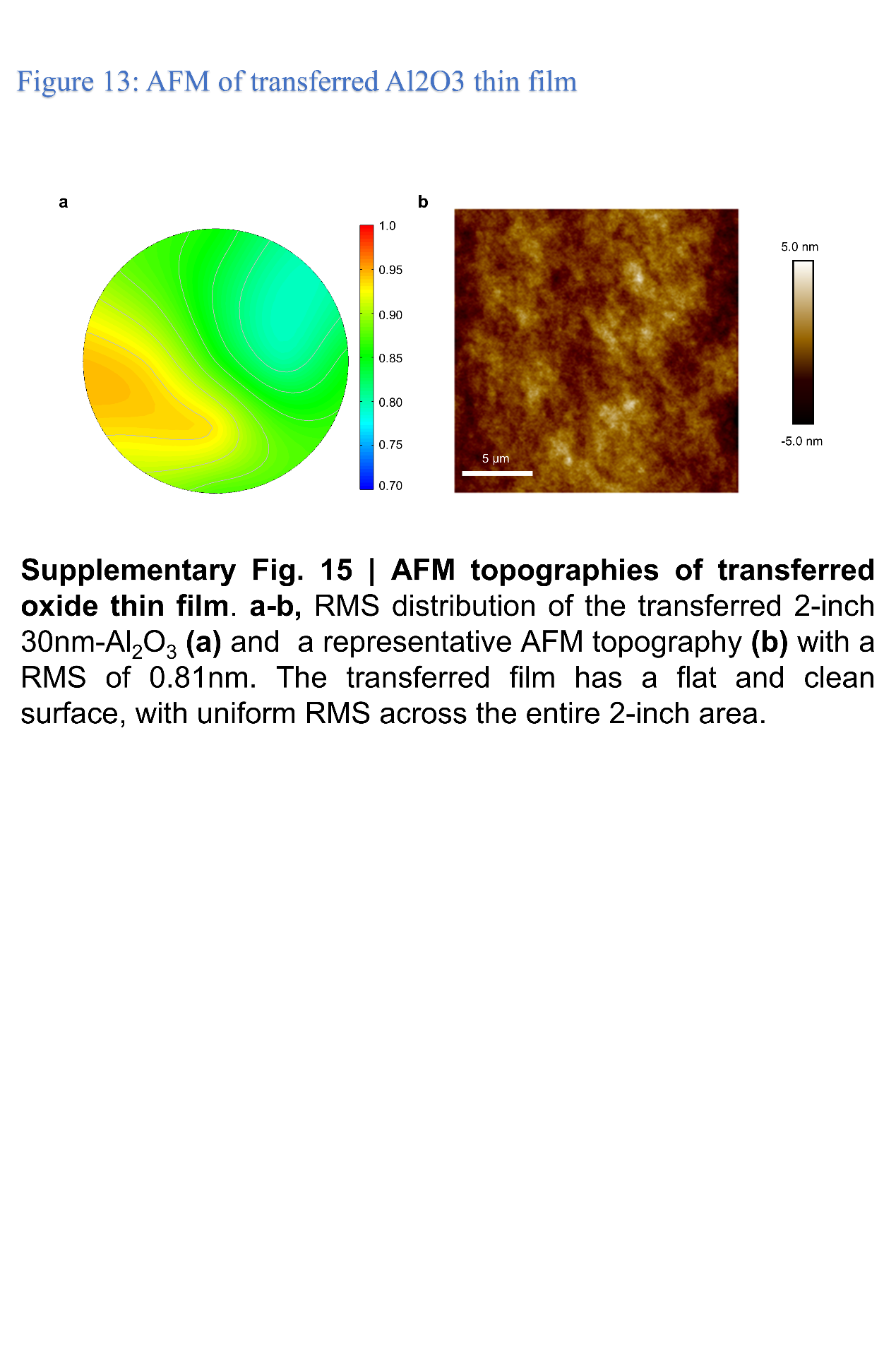


**Figure S18**. AFM topographies of transferred oxide thin film. a-b) RMS distribution of a transferred 2-inch 30nm Al_2_O_3_ (a) and a representative AFM topography (b) with an RMS of 0.81nm. The transferred film has a flat and clean surface, with uniform RMS across the entire 2-inch area.


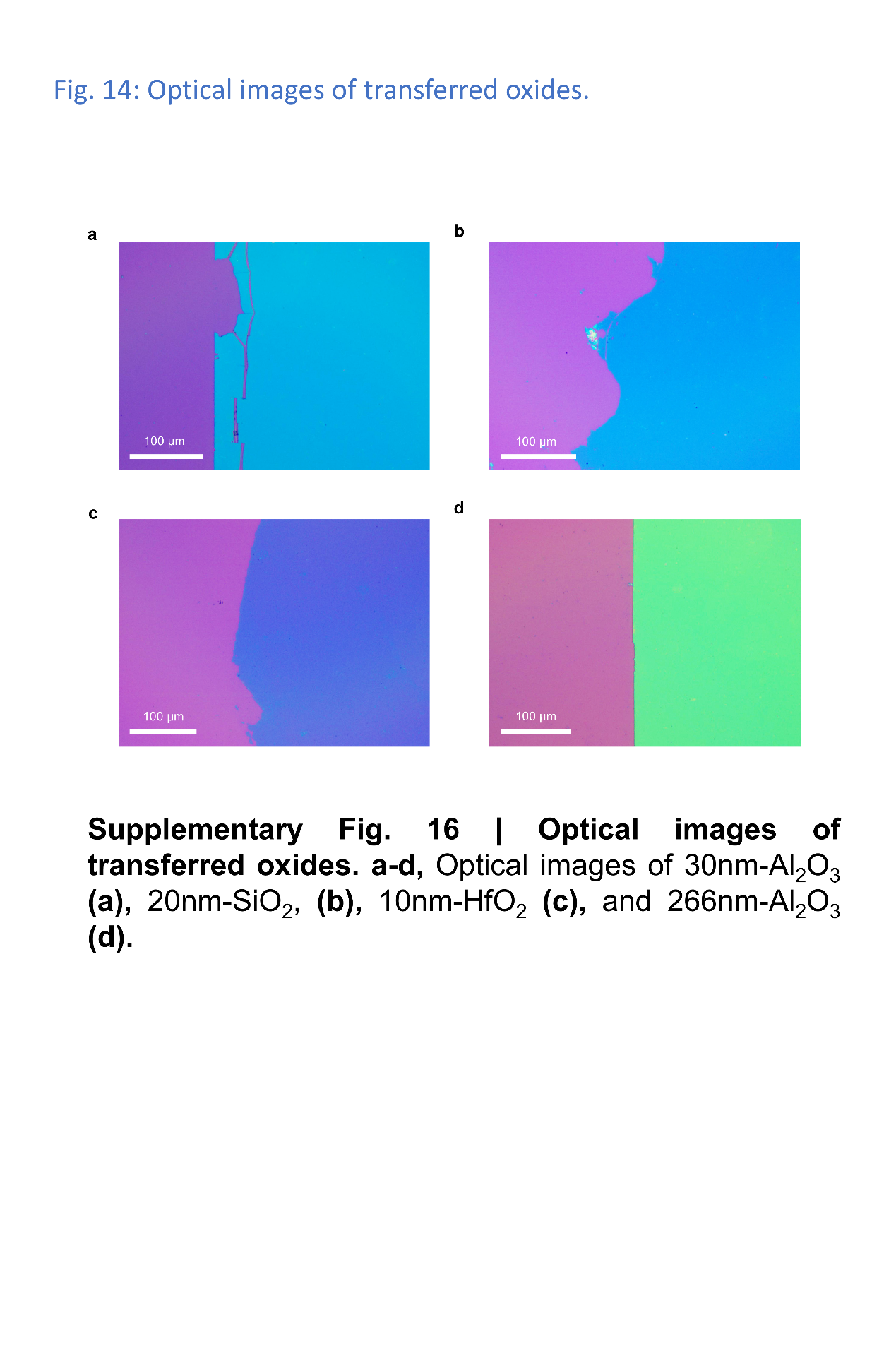


**Figure S19.** Optical images of transferred oxides. a-d), Optical images of 30nm Al_2_O_3_ (a), 20nm SiO_2_, (b), 10nm HfO_2_ (c), and 266nm Al_2_O_3_ (d). The substrates are SiO_2_/Si.

# **Nore S5.** Characterizations of ALD deposited Al_2_O_3_ thin film on MoS_2_.

Al_2_O_3_ layers with intended thickness of 2nm, 5nm, and 10nm were deposited on the MoS_2_ and Si using ALD. The AFM topographies of these deposited Al_2_O_3_ layers are displayed in Figure S20. Compared to Si substrate, Al_2_O_3_ film fails to form on MoS_2_ with low number of deposition cycles (Figure S20a) since the lack of dangling bond on 2D surface. An incomplete film with the thickness of approximately 5nm forms when the number of deposition cycles increases. Further increasing the deposition cycles results in the formation of a 10 nm film with a smooth converged surface. In contrast, 2nm Al_2_O_3_ film with smooth surface can form on Si. The dielectric constants of the films were measured (Al_2_O_3_ deposited on MoS_2_ were transferred to a Si chip for characterization). The results show similar dielectric constants for Al_2_O_3_ deposited on MoS_2_ and Si at comparable thicknesses.


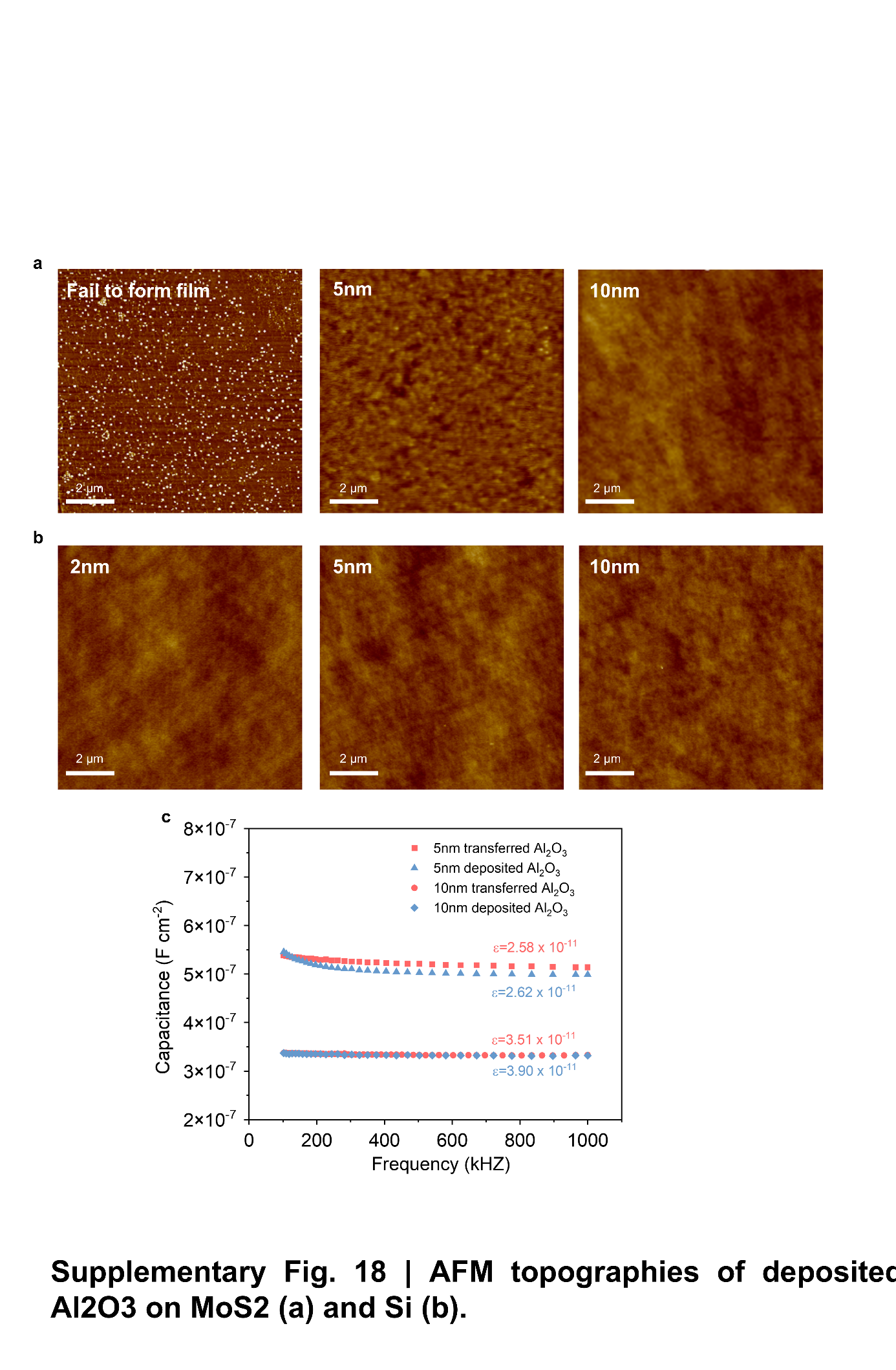


**Figure S20**. Characterizations of transferred Al_2_O_3_. a) AFM topographies of ALD deposited Al_2_O_3_ on MoS_2_ with intended thickness of 2 nm (left), 5 nm (middle), and 10 nm (right). The real thickness was measured to be 0 nm, 4.9 nm and 10.5 nm respectively using an ellipsometer integrated in ALD equipment. b) AFM topographies of ALD deposited Al_2_O_3_ on Si with intended thickness of 2 nm (left), 5 nm (middle), and 10 nm (right). The real thickness was measured to be 2.2 nm, 5.2 nm and 12 nm respectively. c) Capacity as a function of frequency.


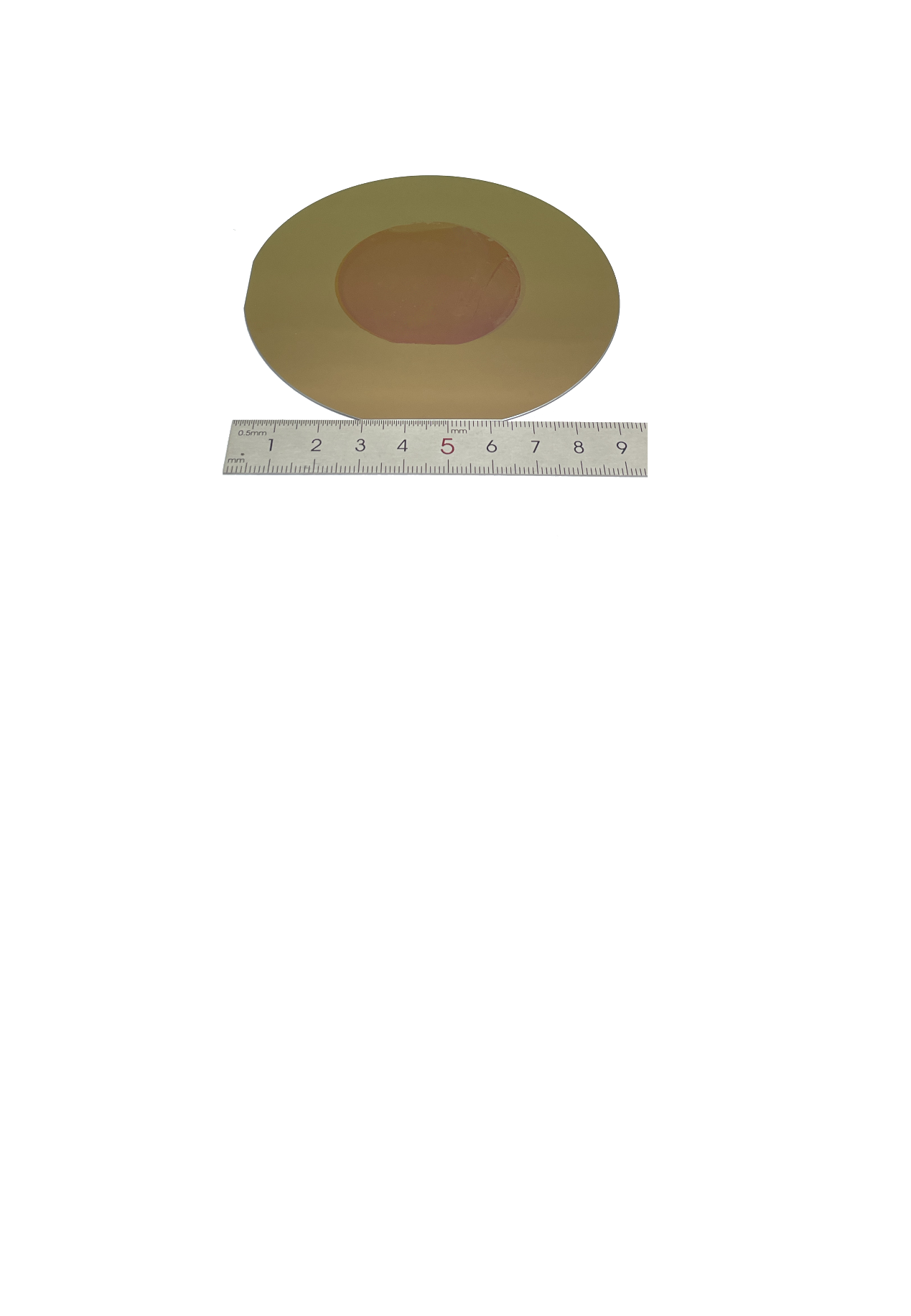


# **Figure S21.** Photograph of a 2-inch MoS_2_/Al_2_O_3_ heterostructure on SiO_2_/Si.

# **Note S6.** Calculation of coherence length.

When the crystal thickness *d* is less than or equal to the coherence length *ΔL (d ≤ ΔL)*, the nonlinear behavior can be described by the relationship *I_SHG_ ∝ d^2^* (where *I_SHG_* is the intensity of SHG). For thicker crystals *(d > ΔL)*, I_SHG_ ​stops increasing and begins to decrease. In reflection geometry, the coherence length is given by the following equation:^[29-30]^

$$\begin{aligned} \Delta L=\frac{\lambda_{\omega}}{4\left( n_{\omega}+n_{2\omega} \right)}\#\left( 1 \right) \end{aligned}$$

where *λ_ω_* is the fundamental excitation wavelength, *n_ω_* = 4.4 and *n_2ω_* = 5.6 refer to refractive indices at fundamental and SHG wavelength respectively^[31]^. With the fundamental wavelength of 1064nm, this gives *∆L* of approximately 26 nm.

# **Note S7.** Calculation detail about SHG intensity in 2D/3D superlattice.

Figure S22 shows SHG process for the bilayer MoS_2_ and trilayer system with oxides. The fundamental excitation laser (red vector) irradiates the materials vertically and two SH signals are excited in the upper (SH1, yellow vector) and lower 1L-MoS_2_ (SH2, blue vector) and are reflected at the SiO_2_/Si interface. To simplify the model, the influence from MoS_2_/Al_2_O_3_ interface was neglected. The consistency between the experimental results and the calculated results (Figure 4) validated the feasibility of the model.

For bilayer MoS_2_ without oxide thin film as shown in Supplementary Figure 22a (top), if the laser electric field $\vec{E}_{2\omega}$ makes an angle of $\phi_{1}(\phi_{2})$ with respect to the armchair direction of top layer (bottom layer), electric field of SH response from top and bottom layer could be described as $\vec{E}_{2\omega}\left( SH1' \right)$ and $\vec{E}_{2\omega}\left( SH2' \right)$:^[32]^

$$\begin{aligned} E_{2\omega}\left( SH1^{'} \right)=\sqrt{I_{1}}e^{i\left( 2\omega t-3\phi_{1} \right)}\#\left( 2 \right) \end{aligned}$$

$$\begin{aligned} E_{2\omega}\left( SH2^{'} \right)=\sqrt{I_{2}}e^{i\left( 2\omega t-3\phi_{2} \right)}\#\left( 3 \right) \end{aligned}$$

The total electric field of SH response obtained by a vector superposition $\vec{E'}$ = $\vec{E}_{2\omega}\left( SH1' \right)$+$\vec{E}_{2\omega}\left( SH2' \right)$. And the total intensity of the stacking region could be calculated by following formular:^[32]^

$$\begin{aligned} I^{'}\propto\left| \vec{E^{'}} \right|^{2}=\left( \left| \vec{E}_{2\omega}\left( SH1^{'} \right) \right|+\left| \vec{E}_{2\omega}\left( SH2^{'} \right) \right| \right)^{2}=I_{1}+I_{2}+2\sqrt{I_{1}I_{2}}\cos3\theta\#\left( 4 \right) \end{aligned}$$

$$\begin{aligned} \theta=\phi_{1}-\phi_{2}\#\left( 5 \right) \end{aligned}$$

A schematic for illustrating the vector superposition of the SH electric fields was shown in Figure S22a (bottom).

With oxide thin film as shown in Supplementary Figure S22b (top), oxides induce an optical path difference and an extra phase difference *ψ*. Thus, $\vec{E}_{2\omega}\left( SH1' \right)$ and $\vec{E}_{2\omega}\left( SH2' \right)$ were replaced by $\vec{E}_{2\omega}\left( SH1 \right)$ and $\vec{E}_{2\omega}\left( SH2 \right)$ as following equation:

$$\begin{aligned} E_{2\omega}\left( SH1 \right)=\sqrt{I_{1}}e^{i\left( 2\omega t-3\phi_{1}-\psi\right)}\#\left( 6 \right) \end{aligned}$$

$$\begin{aligned} E_{2\omega}\left( SH2 \right)=E_{2\omega}\left( SH2^{'} \right)=\sqrt{I_{2}}e^{i\left( 2\omega t-3\phi_{2} \right)}\#\left( 7 \right) \end{aligned}$$

$$\begin{aligned} \psi=\frac{2d\pi}{\lambda_{2\omega}}\#\left( 8 \right) \end{aligned}$$

The total SHG intensity of the stacking region with inserted oxides could be calculated by following formular:

$$\begin{aligned} I\propto\left| \vec{E} \right|^{2}=\left( \left| \vec{E}_{2\omega}\left( SH1 \right) \right|+\left| \vec{E}_{2\omega}\left( SH2 \right) \right| \right)^{2}=I_{1}+I_{2}+2\sqrt{I_{1}I_{2}}\cos\left( 3\theta-\psi\right)\#\left( 9 \right) \end{aligned}$$

A schematic for illustrating the vector superposition of the SH electric fields is shown in Figure S22b (bottom). The condition for maximizing intensity of SHG is as follow:

$$\begin{aligned} 3\theta-\psi=0\#\left( 10 \right) \end{aligned}$$

Phase mismatch arises from the phase differences accumulated when fundamental wave travel across a thick material. In our approach, QPM is achieved by modulating the phase difference through controlled twist angles and the optical path difference introduced by inserted oxides.


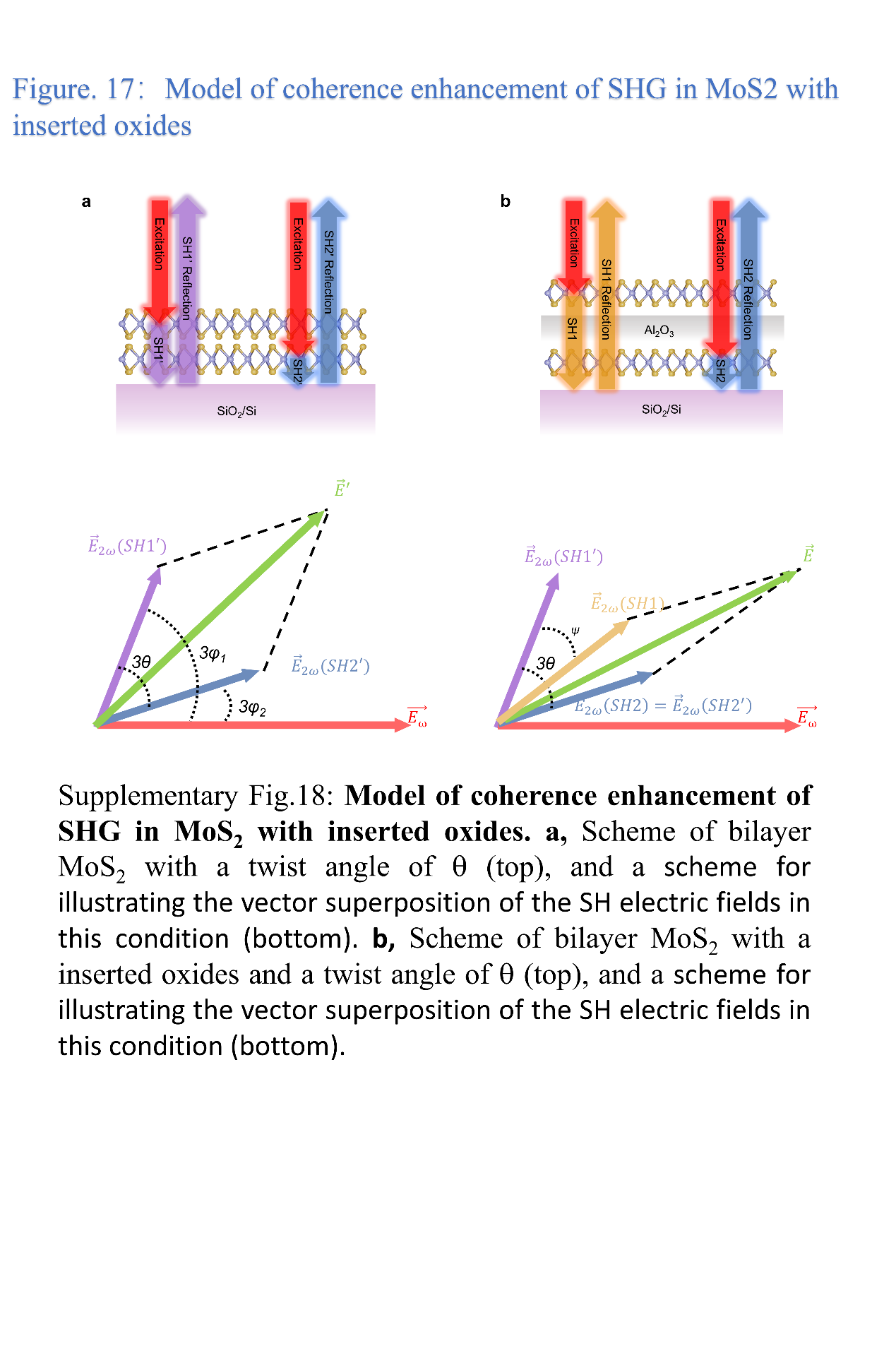


**Figure S22.** Model of coherence enhancement of SHG in MoS_2_ with inserted oxides. a), Schematic of generation and propagation of SH signals in bilayer MoS_2_ with a twist angle of q (top), and a schematic for illustrating the vector superposition of the SH electric fields in this condition (bottom). b), Schematic of the generation and propagation of SH signals in oxide/MoS_2_ superlattice with an inserted oxides and a twist angle of q (top), and a schematic for illustrating the vector superposition of the SH electric fields in this condition (bottom).


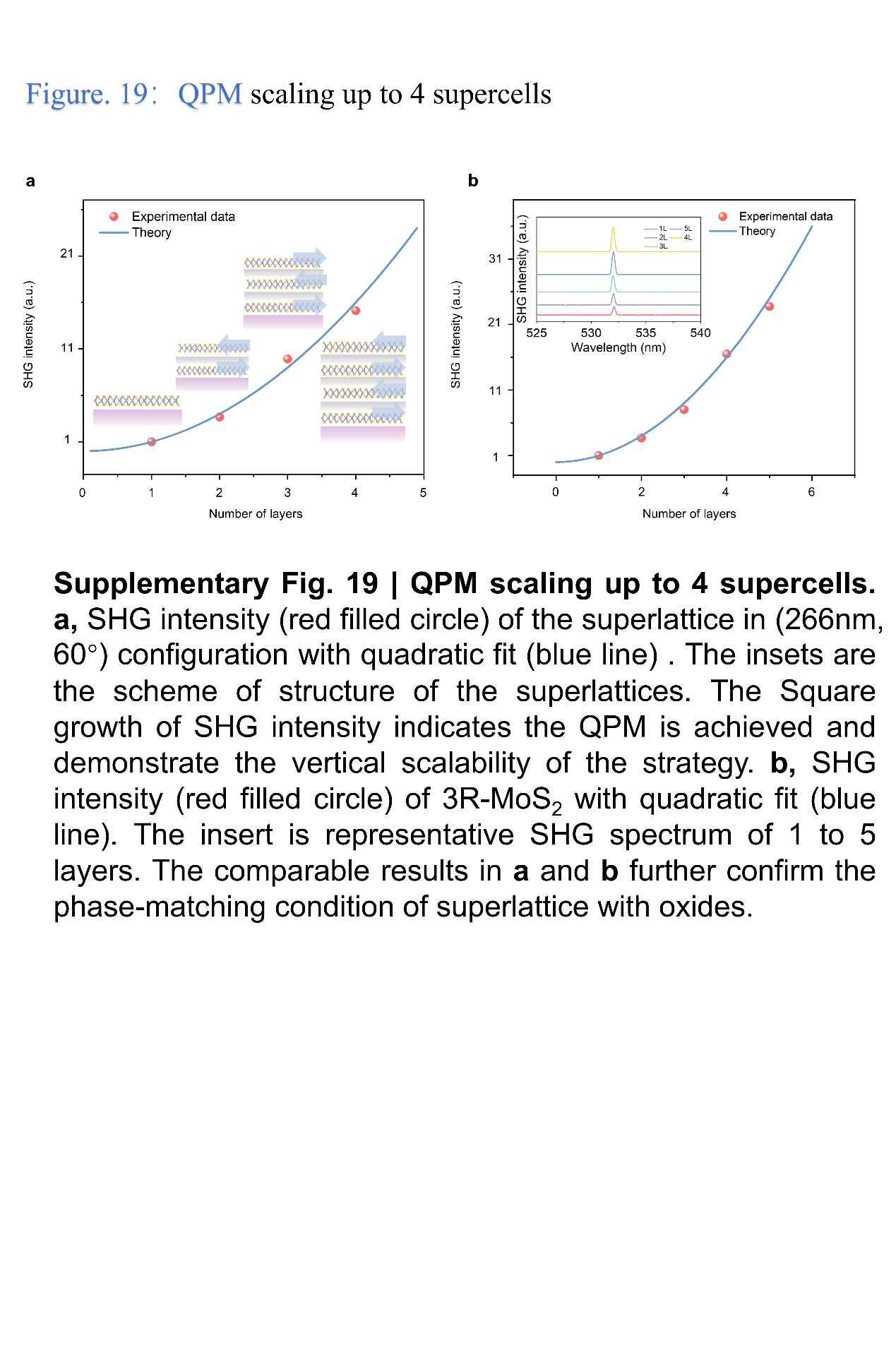


**Figure S23.** QPM condition scaling up to three supercells. a), SHG intensity (red filled circles) of the superlattice with an interlayer Al₂O₃ thickness of 266 nm and twist angles of 60° between adjacent MoS₂ layers, along with a quadratic fit (blue line). The insets show schematic structures of the superlattices with different layer numbers. The square growth of SHG intensity indicates that QPM is achieved. b) SHG intensity (red filled circles) of a phase-matched stacked 3R-MoS₂, with a quadratic fit (blue line). The inset shows representative SHG spectra from 1 to 5 layers. The comparable results in a and b further confirm the QPM condition of the 2D/3D superlattice.


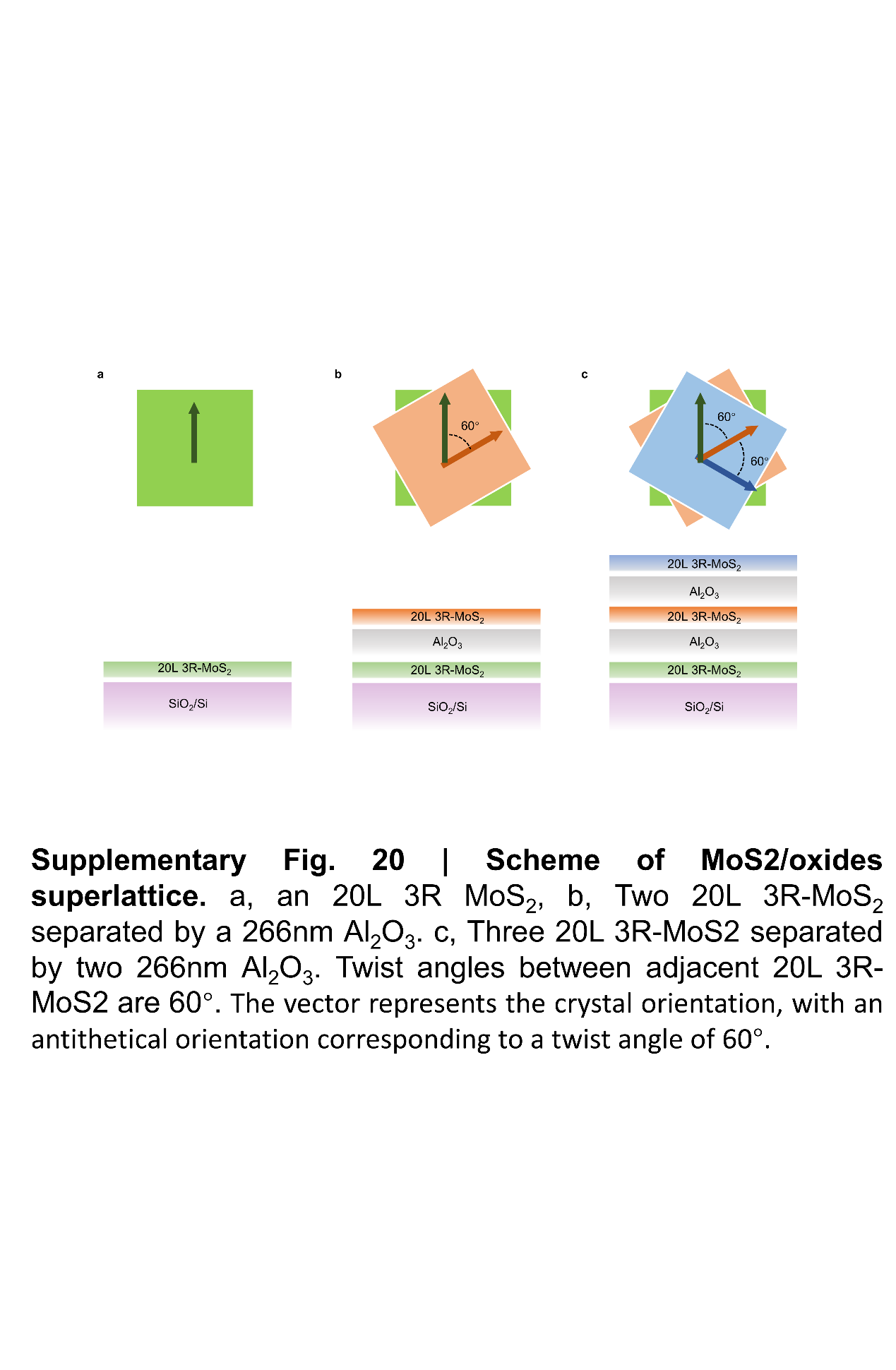


**Figure S24**. Scheme of MoS_2_/oxides superlattice in Figure 4d. a) an 20L 3R MoS_2_, b) 40L 3R-MoS_2_ separated by a 266nm Al_2_O_3_, c) 60L 3R-MoS_2_ separated by two 266nm Al_2_O_3_. Twist angles between adjacent 20L 3R-MoS2 are 60°.

# **Note S8.** QPM condition with ML 3R-MoS_2_/oxide superlattice.

For 2D materials, SHG intensity shows a quadratic relationship with the number of layers under phase-matching condition.^[29, 33]^ In this study, QPM was achieved in 20L 3R-MoS_2_/oxide superlattice (Figure 4d), in which each 20L 3R-MoS_2_ was treated as an individual nonlinear crystal. Consequently, SHG intensity exhibits a quadratic relationship with the number of these nonlinear crystals. Therefore, the total intensity of SHG can be calculated using the following formula:

$$\begin{aligned} I=I_{20L}\left( \frac{N}{20} \right)^{2}\#\left( 11 \right) \end{aligned}$$

Where *I_20L_* SHG intensity of 20L 3R-MoS_2_ and *N* is the number of MoS_2_ layers in the superlattice.


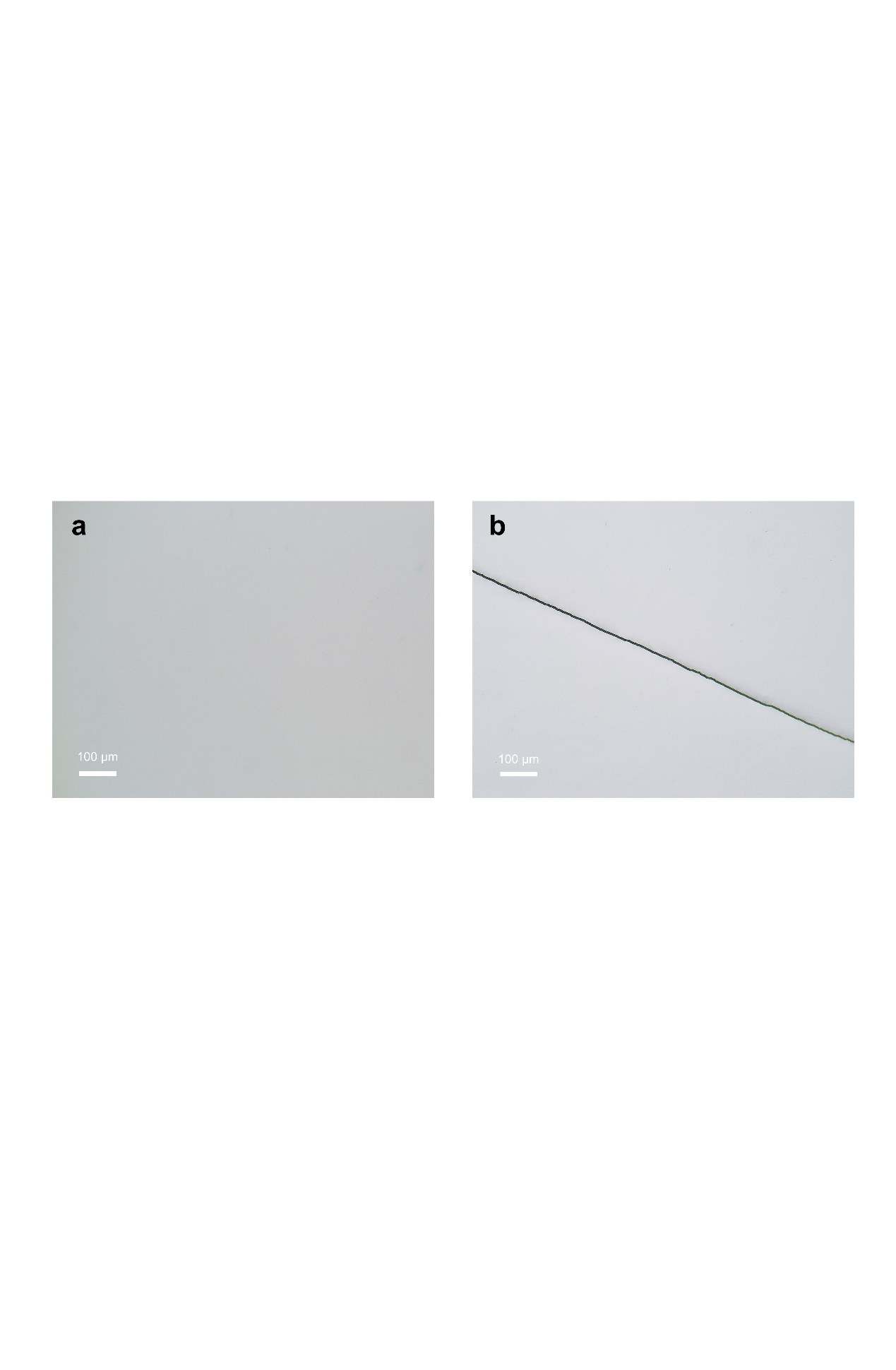


**Figure 25.** Optical images of Cu films after exfoliation process. a-b), Optical images of 200 nm (a) and 50 nm (b) Cu film after exfoliation.

**Reference**

[1] Y. Duo, Q. Yang, L. Wang, Y. Song, Z. Huo, J. Ran, J. Yang, J. Wang, T. J. C. G. Wei, Design, *Cryst. Growth Des.* **2024**, 24, 843.

[2] A. Gurarslan, Y. Yu, L. Su, Y. Yu, F. Suarez, S. Yao, Y. Zhu, M. Ozturk, Y. Zhang, L. Cao, *ACS Nano* **2014**, 8, 11522.

[3] J. H. Kim, T. J. Ko, E. Okogbue, S. S. Han, M. S. Shawkat, M. G. Kaium, K. H. Oh, H. S. Chung, Y. Jung, *Sci. Rep.* **2019**, 9, 1641.

[4] M. Liao, Z. Wei, L. Du, Q. Wang, J. Tang, H. Yu, F. Wu, J. Zhao, X. Xu, B. Han, K. Liu, P. Gao, T. Polcar, Z. Sun, D. Shi, R. Yang, G. Zhang, *Nat. Commun.* **2020**, 11, 2153.

[5] X. Li, Y. Zhu, Y. Cai, Y. Borysiak, Boyang Han, David Chen, Richard D. Piner, Luigi Colombo, R. S. Ruoff, *Nano Lett.* **2009**, 9, 4359.

[6] S. Bae, H. Kim, Y. Lee, X. Xu, J. S. Park, Y. Zheng, J. Balakrishnan, T. Lei, H. R. Kim, Y. I. Song, Y. J. Kim, K. S. Kim, B. Ozyilmaz, J. H. Ahn, B. H. Hong, S. Iijima, *Nat. Nanotechnol.* **2010**, 5, 574.

[7] J. Shim, S.-H. Bae, W. Kong, D. Lee, K. Qiao, D. Nezich, Y. J. Park, R. Zhao, S. Sundaram, X. Li, *Science* **2018**, 362, 665.

[8] K. Kang, K. H. Lee, Y. Han, H. Gao, S. Xie, D. A. Muller, J. Park, *Nature* **2017**, 550, 229.

[9] S. Boandoh, F. O. Agyapong-Fordjour, S. H. Choi, J. S. Lee, J. H. Park, H. Ko, G. Han, S. J. Yun, S. Park, Y. M. Kim, W. Yang, Y. H. Lee, S. M. Kim, K. K. Kim, *ACS Appl. Mater. Interfaces* **2019**, 11, 1579.

[10] A. J. Mannix, A. Ye, S. H. Sung, A. Ray, F. Mujid, C. Park, M. Lee, J. H. Kang, R. Shreiner, A. A. High, D. A. Muller, R. Hovden, J. Park, *Nat. Nanotechnol.* **2022**, 17, 361.

[11] Y. T. Megra, J. W. Suk, *J. Phys. D: Appl. Phys.* **2019**, 52, 364002.

[12] M. R. Rosenberger, H. J. Chuang, M. Phillips, V. P. Oleshko, K. M. McCreary, S. V. Sivaram, C. S. Hellberg, B. T. Jonker, *ACS Nano* **2020**, 14, 4550.

[13] J. Baek, H. G. Kim, S. Y. Lim, S. C. Hong, Y. Chang, H. Ryu, Y. Jung, H. Jang, J. Kim, Y. Zhang, K. Watanabe, T. Taniguchi, P. Y. Huang, H. Cheong, M. Kim, G. Lee, *Nat. Mater.* **2023**, 22, 1463.

[14] T. Wu, C. Sher, Y. Lin, C. Lee, S. Liang, Y. Lu, Huang Chen, W. Guo, H. Kuo, Z. Chen, *Applied Sciences* **2018**, 8, 1557.

[15] X. Zhou, P. Tian, C. Sher, J. Wu, H. Liu, R. Liu, H. Kuo, *Prog. Quantum Electron.* **2020**, 71, 100263.

[16] A. Jain, P. Bharadwaj, S. Heeg, M. Parzefall, T. Taniguchi, K. Watanabe, L. Novotny, *Nanotechnology* **2018**, 29, 265203.

[17] J. Quan, L. Linhart, M. L. Lin, D. Lee, J. Zhu, C. Y. Wang, W. T. Hsu, J. Choi, J. Embley, C. Young, T. Taniguchi, K. Watanabe, C. K. Shih, K. Lai, A. H. MacDonald, P. H. Tan, F. Libisch, X. Li, *Nat. Mater.* **2021**, 20, 1100.

[18] S. Huang, L. Liang, X. Ling, A. A. Puretzky, D. B. Geohegan, B. G. Sumpter, J. Kong, V. Meunier, M. S. Dresselhaus, *Nano Lett.* **2016**, 16, 1435.

[19] K. Liu, L. Zhang, T. Cao, C. Jin, D. Qiu, Q. Zhou, A. Zettl, P. Yang, S. G. Louie, F. Wang, *Nat. Commun.* **2014**, 5, 4966.

[20] A. Daus, S. Vaziri, V. Chen, Ç. Köroğlu, R. W. Grady, C. S. Bailey, H. R. Lee, K. Schauble, K. Brenner, E. Pop, *Nat. Electron.* **2021**, 4, 495.

[21] A. Dodda, D. Jayachandran, A. Pannone, N. Trainor, S. P. Stepanoff, M. A. Steves, S. S. Radhakrishnan, S. Bachu, C. W. Ordonez, J. R. Shallenberger, J. M. Redwing, K. L. Knappenberger, D. E. Wolfe, S. Das, *Nat. Mater.* **2022**, 21, 1379.

[22] N. Li, Q. Wang, C. Shen, Z. Wei, H. Yu, J. Zhao, X. Lu, G. Wang, C. He, L. Xie, J. Zhu, L. Du, R. Yang, D. Shi, G. Zhang, *Nat. Electron.* **2020**, 3, 711.

[23] X. Xu, C. Trovatello, F. Mooshammer, Y. Shao, S. Zhang, K. Yao, D. N. Basov, G. Cerullo, P. J. Schuck, *Nat. Photonics* **2022**, 16, 698.

[24] I. Abdelwahab, B. Tilmann, Y. Wu, D. Giovanni, I. Verzhbitskiy, M. Zhu, R. Berté, F. Xuan, L. d. S. Menezes, G. Eda, T. C. Sum, S. Y. Quek, S. A. Maier, K. P. Loh, *Nat. Photonics* **2022**, 16, 644.

[25] A. J. Yang, K. Han, K. Huang, C. Ye, W. Wen, R. Zhu, R. Zhu, J. Xu, T. Yu, P. Gao, Q. Xiong, X. Renshaw Wang, *Nat. Electron.* **2022**, 5, 233.

[26] J. Huang, Y. Wan, J. Shi, J. Zhang, Z. Wang, W. Wang, N. Yang, Y. Liu, C. Lin, X. Guan, L. Hu, Z. Yang, B. Huang, Y. Chiu, J. Yang, V. Tung, D. Wang, K. Kalantarzadeh, T. Wu, X. Zu, L. Qiao, L. Li, S. Li, *Nature* **2022**, 605, 262.

[27] M. Sebek, Z. Wang, N. G. West, M. Yang, D. C. J. Neo, X. Su, S. Wang, J. Pan, N. T. K. Thanh, J. Teng, *npj 2D Mater. Appl.* **2024**, 8, 9.

[28] Z. Lu, Y. Chen, W. Dang, L. Kong, Q. Tao, L. Ma, D. Lu, L. Liu, W. Li, Z. Li, X. Liu, Y. Wang, X. Duan, L. Liao, Y. Liu, *Nat. Commun.* **2023**, 14, 2340.

[29] J. Shi, P. Yu, F. Liu, P. He, R. Wang, L. Qin, J. Zhou, X. Li, J. Zhou, X. Sui, S. Zhang, Y. Zhang, Q. Zhang, T. C. Sum, X. Qiu, Z. Liu, X. Liu, *Adv. Mater.* **2017**, 29, 1701486.

[30] E. Mishina, N. Sherstyuk, S. Lavrov, A. Sigov, A. Mitioglu, S. Anghel, L. Kulyuk, *Appl. Phys. Lett.* **2015**, 106, 131901.

[31] V. Sobolev, V. V. Sobolev, *Journal of Applied Spectroscopy* **1994**, 61, 532.

[32] W. Hsu, Z. Zhao, L. Li, C. Chen, M. Chiu, P. Chang, Y. Chou, W. Chang, *ACS Nano* **2014**, 8, 2951.

[33] F. Liu, W. J. Wu, Y. S. Bai, S. H. Chae, Q. Y. Li, J. Wang, J. Hone, X. Y. Zhu, *Science* **2020**, 367, 903.
